# Functional Consequences of the Evolution of Matrimony, a Meiosis-Specific Inhibitor of Polo Kinase

**DOI:** 10.1093/molbev/msy197

**Published:** 2018-10-23

**Authors:** Amanda M Bonner, R Scott Hawley

**Affiliations:** 1 Stowers Institute for Medical Research, Kansas City, MO; 2 Department of Molecular and Integrative Physiology, University of Kansas Medical Center, Kansas City, KS

**Keywords:** meiosis, positive selection, molecular evolution, Polo kinase, *Drosophila*

## Abstract

Meiosis is a defining characteristic of eukaryotes, believed to have evolved only once, over one billion years ago. While the general progression of meiotic events is conserved across multiple diverse organisms, the specific pathways and proteins involved can be highly divergent, even within species from the same genus. Here we investigate the rapid evolution of Matrimony (Mtrm), a female meiosis-specific regulator of Polo kinase (Polo) in *Drosophila*. Mtrm physically interacts with Polo and is required to restrict the activity of Polo during meiosis. Despite Mtrm’s critical role in meiosis, sequence conservation within the genus *Drosophila* is poor. To explore the functional significance of this rapid divergence, we expressed Mtrm proteins from 12 different *Drosophila* species in the *Drosophila melanogaster* female germline. Distantly related Mtrm homologs are able to both physically interact with *D. melanogaster* Polo and rescue the meiotic defects seen in *mtrm* mutants. However, these distant homologs are not properly degraded after the completion of meiosis. Rather, they continue to inhibit Polo function in the early embryo, resulting in dominant maternal-effect lethality. We show that the ability of Mtrm to be properly degraded, and thus release Polo, is partially due to residues or motifs found within Mtrm’s least-conserved regions. We hypothesize that, while Mtrm regions critical for its meiotic function are under strong purifying selection, changes that occurred in its unconserved regions may have been advantageous, potentially by affecting the timing or duration of meiosis and/or the early embryonic divisions.

## Introduction

The progression of cell cycle events is tightly regulated by the controlled oscillations of expression, activation, and/or degradation of key cell cycle regulators—for example, cyclin-dependent kinases, cyclins, and the E3 ubiquitin ligase known as the anaphase-promoting complex/cyclosome (APC/C)—many of which are highly conserved across all eukaryotes. Meiosis is a variant cell division in which DNA replication is followed by two rounds of chromosome segregation with no intervening S phase, resulting in haploid gametes (Marston and Amon 2004; Gerton and Hawley 2005). While cells undergoing meiosis utilize much of the mitotic machinery, they require additional regulation to properly progress through the meiotic program.

One family of proteins involved in cell cycle progression are the Polo-like kinases (Plks). First characterized in *Drosophila melanogaster*, Plks are conserved from yeast to mammals and are often referred to as master regulators of the cell cycle, as they play multiple roles in controlling cell division in both mitosis and meiosis (Sunkel and Glover 1988; Llamazares et al. 1991; Archambault and Glover 2009). With the multiple functions Plks play during cell division, it is not surprising that the regulation of Plks is a critical cellular process. In *D. melanogaster* females, Polo kinase (Polo) must be inhibited during the first meiotic division. This inhibition is achieved by Polo’s interaction with the female meiosis-specific Matrimony (Mtrm) protein.

The *mtrm* gene is a small, intronless gene that is highly expressed only in the ovary (Xiang et al. 2007). Mtrm function is critical during female meiosis, where it binds to and inhibits Polo (Xiang et al. 2007). The *mtrm* gene is haploinsufficient, and heterozygous females that possess a single functional copy of *mtrm* display high levels of missegregation of achiasmate chromosomes during the first meiotic division (Harris et al. 2003). That missegregation can be rescued, however, if females are simultaneously heterozygous for a null mutation of *polo* (Xiang et al. 2007). Nonfunctional *mtrm* mutant alleles also induce precocious breakdown of the oocyte nuclear envelope, as both hetero- and homozygotes (Xiang et al. 2007). In *mtrm*/+ heterozygotes, as is true for the chromosome missegregation phenotype, the early nuclear envelope breakdown phenotype is suppressible by simultaneous reduction of the dosage of the *polo* gene (Xiang et al. 2007). When females carry no functional copies of *mtrm*, their observed phenotypes are much more severe, including chromosome fragmentation during meiosis I, cessation of the meiotic process, and sterility (Bonner et al. 2013).

Mtrm protein levels, which increase during meiosis I (Von Stetina et al. 2011), are significantly decreased upon completion of meiosis II, allowing Polo to be active in the early embryonic divisions (Whitfield et al. 2013). Degradation of Mtrm at the meiosis-to-mitosis transition is critical for proper development of the early embryo and requires the activity of Cortex (Whitfield et al. 2013), a female meiosis-specific activator of the APC/C (Chu et al. 2001; Pesin and Orr-Weaver 2007). Mutants that prevent the timely degradation of Mtrm result in embryonic developmental defects, presumably arising as a consequence of the absence of active Polo (Whitfield et al. 2013).

**Fig. 1.**
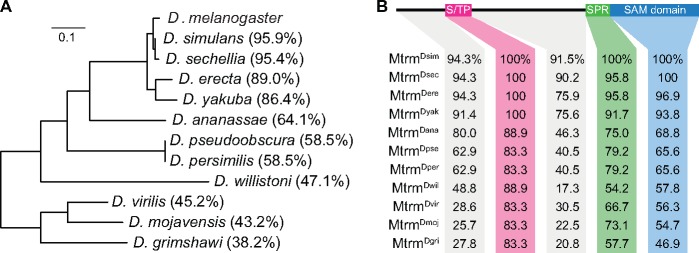
Conservation of the Mtrm protein in 12 *Drosophila* species. (*A*) Phylogenetic tree of *mtrm* sequences constructed using IQ-TREE. Scale bar shows number of nucleotide substitutions per site. Numbers in parentheses show total percent protein identity of corresponding proteins, compared with Mtrm^Dmel^. (*B*) Percent protein identity of Mtrm homologs by region, compared with Mtrm^Dmel^.

Given Mtrm’s role as a critical regulator of the highly conserved Polo, one might assume that the *mtrm* gene would also be highly conserved. On the contrary, *mtrm* homolog sequences are quite divergent even within the *Drosophila* genus (fig. 1*A* and *B*). In fact, previous analysis has demonstrated that the *mtrm* gene shows a higher-than-expected number of fixed nonsynonymous changes between the closely related *D. melanogaster* and *D. simulans* species, indicative of positive selection affecting its evolution (Anderson et al. 2009).

We therefore wanted to investigate the functional significance of the divergence of *mtrm* sequence by expressing *mtrm* homologs from increasingly divergent *Drosophila* species in *D. melanogaster* females. We show that expression of all *Drosophila mtrm* homologs can rescue the meiotic phenotypes observed in *mtrm* mutant backgrounds, supporting the idea that the conserved regions of the Mtrm protein are under strong purifying, or negative, selection, as they are critical for its meiotic function. Interestingly, we observe that Mtrm’s poorly conserved regions are also able to affect its function by altering the protein’s stability and/or its ability to be degraded at the proper time. We also provide additional evidence that a signature of positive selection exists for *mtrm*, at least for homologs within the *melanogaster* group, though only for its highly divergent central region, suggesting that nonsynonymous changes between species occurring in that region could be advantageous. Together, these data provide functional evidence to support our evolutionary analyses showing that different regions of the *mtrm* gene are under very different selective pressures.

## Results

### Mtrm Homologs from 12 *Drosophila* Species Are Highly Divergent

Within the *Drosophila* genus, few *mtrm* homologs outside of *D. melanogaster* have been annotated. However, the *mtrm* gene can reliably be found within an intron of the *exo70* gene, as this synteny is conserved for all orthologs from the 12 *Drosophila* species sequenced by the Drosophila 12 Genomes Consortium (2007). In addition, two independent duplications of the *mtrm* gene have been reported, resulting in paralogs in both *D. willistoni* and *D. virilis* (Reis et al. 2011). The phylogenetic tree created from *mtrm* sequence alignments corresponds with the species’ current accepted phylogeny (Drosophila 12 Genomes Consortium 2007) (fig. 1*A*). However, overall sequence conservation among the 12 *Drosophila* species is poor, as Mtrm sequences from *D. melanogaster* and *D. grimshawi*, which shared a common ancestor over 60 Ma (Tamura et al. 2004), share only 38.2% protein sequence identity (fig. 1*A*). This is well below the average protein identity of 72.4% between *D. melanogaster* and *D. grimshawi*, based on protein alignments provided by flyDIVaS of genes for which there are homologs in all 12 *Drosophila* species (Stanley and Kulathinal 2016).

Despite the low sequence identity shared between distantly related Mtrm orthologs, the protein does contain three blocks of conservation (fig. 1*B* andsupplementary fig. S1, Supplementary Material online). The first is a 19-amino acid-long region near Mtrm’s N-terminus that contains three phosphorylated residues we have previously shown to be required for *D. melanogaster* Mtrm’s interaction with Polo (Xiang et al. 2007; Bonner et al. 2013). We will refer to this as the S/TP region, based on its sequence, as it contains three pS/pT-P motifs. At Mtrm’s C-terminus is a sterile alpha motif (SAM) domain that stabilizes the Mtrm::Polo interaction (Bonner et al. 2013). There is also a stretch of conserved residues just proximal to the SAM domain we will refer to as the SAM-proximal region.

Mtrm protein length is variable among the 12 *Drosophila* species, ranging from 191 amino acids in *D. mojavensis* to 219 amino acids in *D. yakuba*, a difference of 13.6% (supplementary fig. S1, Supplementary Material online). The vast majority of this variation in length is found within Mtrm’s N-terminal and central unconserved regions, which together make up approximately half of the protein length. As evolutionary distance between *mtrm* homologs increases, the level of sequence identity in those unconserved regions decreases, as does the ability to align those regions properly, as assayed by GUIDANCE2, a methodology that assigns a confidence score for each column in a multiple sequence alignment (MSA) (Sela et al. 2015) (supplementary fig. S1, Supplementary Material online).

### Mtrm’s Different Regions Are Evolving at Different Rates

**Fig. 2.**
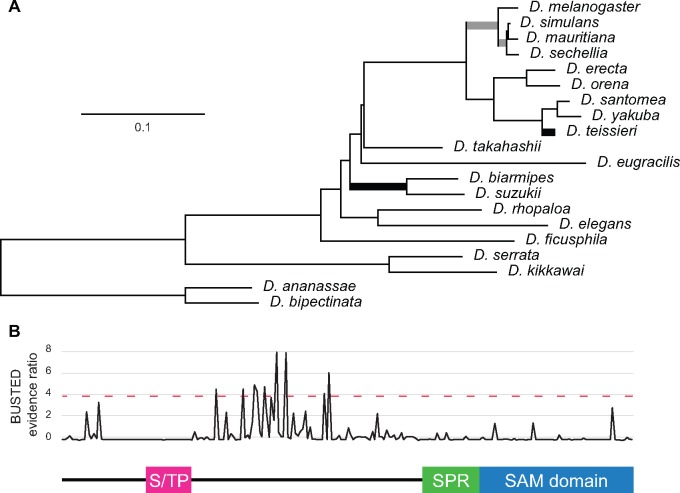
Branch-site analyses of *mtrm* from the *melanogaster* group. (*A*) Phylogenetic tree of *mtrm* from 20 *melanogaster* group species, constructed using IQ-TREE. Scale bar shows number of nucleotide substitutions per site. Thick branches correspond to those showing evidence of positive selection (*P *<* *0.05) according to aBSREL. Thick branches in black are those still significant after correcting for multiple testing. (*B*) BUSTED analysis of sites showing evidence of episodic positive selection. Above dashed line corresponds to *P* value < 0.05.

Previous evidence using the McDonald–Kreitman (MK) test (McDonald and Kreitman 1991) to compare nucleotide sequences among 31 *D. melanogaster* and six *D. simulans* lines has suggested that *mtrm* has evolved under positive selection, with an excess of nonsynonymous changes fixed between species (Anderson et al. 2009). To further investigate the selective pressures acting on *mtrm*, we first used codon-based maximum-likelihood methods implemented in the *codeml* program from the PAML suite (Yang 1997) to estimate variation in ω, the ratio of the numbers of nonsynonymous (d*N*) and synonymous (d*S*) substitutions per site. As d*S* can become saturated over large phylogenetic distances, we limited our analyses to species in the *melanogaster* group (fig. 2*A*). We then applied the following random-site models: M7, which assumes a beta distribution of ω over the alignment but constrains ω to values ≤1; and M8, which is similar to M7 but allows an extra site class for ω values >1 (Yang and Swanson 2002). When looking at the MSA for 20 species from the *melanogaster* group, we did not find evidence of positive selection affecting *mtrm* sequence evolution when comparing models M7 and M8 (table 1).

**Table 1.** **PAML Analyses of *mtrm* in the *melanogaster* Group Homologs**.

Random-site models			
Null model	Alternate model	ω^a^	*P* value
M7	M8	ω_0_ = 0.140 (*p*_0_ > 99%)	1.00
		ω_1_ = 3.111 (*p*_1_ < 1%)	
Fixed-site models			
Null model	Alternate model	Parameters^b^	*P* value
A	B	κ = 1.495	*6.90E-16*
		ω = 0.105	
B	D		*5.46E-10*
B	D2^c^	ω_Nterm_ = 0.117	*1.69E-12*
		ω_S/TP_ = 0.010	
		ω_Central_ = 0.171	
		ω_SPR_ = 0.070	
		ω_SAM_ = 0.075	** **

Note.—Individual species used are listed in supplementary table S1, Supplementary Material online, and depicted in figure 2*A*. κ, transition/transversion ratio.

a ω values from alternate model.

b Parameters from alternate model.

c Same as model D, but with κ set to 1.495.

Because of the variability of conservation across the different regions of *mtrm*, we also applied fixed-site models to our MSA, which was partitioned into five sections corresponding to the regions depicted in figure 1*B*. Fixed-site models explore whether different sites/regions in the sequence are under different selective pressures. Specifically, we compared the following models: model A, which assumes a single ω ratio for the entire sequence; model B, which assumes different substitution rates among the different regions; and model D, which assumes different substitution rates, ω values, and transition/transversion ratios (κ) among the regions. Model B fit the data significantly better than model A (*P *<* *0.001), suggesting that the different regions of *mtrm* are evolving at different rates (table 1). Additionally, model D was significantly better than model B (*P *<* *0.001), even when we did not allow κ to vary, suggesting that the ω values among the regions are significantly different from each other (table 1). However, the ω values for each region were <1, implying a role of purifying selection in *mtrm* evolution.

If genes are subjected to episodic adaptive selection, they may not show a signature of positive selection over an entire phylogeny. Therefore, we next examined our MSAs using two different branch-site models, aBSREL (Smith et al. 2015) and BUSTED (Murrell et al. 2015), which allow ω to vary not only over different sites but also episodically over individual branches in the phylogeny. For both models, we made no a priori hypotheses about which branches might be under selection and therefore ran the analyses over the entire *melanogaster* group phylogeny. The aBSREL analysis provided evidence of four branches having undergone episodic positive selection, although only two remained significant (at *P* value < 0.05) after correcting for multiple testing (fig. 2*A*). BUSTED also found evidence of episodic diversifying selection over the phylogeny (*P *<* *0.001), with the sites showing the strongest evidence of positive selection residing in *mtrm’*s central region (fig. 2*B*).

To examine more closely patterns of selection at the different regions of *mtrm*, we next repeated the MK test on *mtrm’*s entire coding sequence as well as region by region, comparing 143 *D. melanogaster* to 28 *D. simulans* lines (table 2 and supplementary tables S2 and S3 and supplementary data 1, Supplementary Material online). The MK test, which compares synonymous and nonsynonymous changes that are either polymorphic or fixed between two closely related species, is used to test the hypothesis that patterns of divergence (in both synonymous and nonsynonymous sites) is predicted by patterns of polymorphism. An excess of nonsynonymous divergence (compared with nonsynonymous polymorphism) is evidence for positive selection (McDonald and Kreitman 1991). Polymorphism and divergence data can also be used to calculate α, or the proportion of adaptive substitutions (Smith and Eyre-Walker 2002). Likewise, these data can also be used to calculate the direction of selection (DoS). Positive DoS values reflect evidence of adaptive or positive selection, while negative values indicate purifying selection (Stoletzki and Eyre-Walker 2011). Because very low-frequency variants are likely to be deleterious mutations that would not be maintained within or among species and can bias the results of the MK test (Messer and Petrov 2013), we excluded any polymorphisms that were present in fewer than 5% of the lines from either species.

**Table 2.** **McDonald–Kreitman Tests for *mtrm* within*D. melanogaster* and *D. simulans***.

							Polarized	
Region	d*N*	d*S*	p*N*	p*S*		Unpolarized	*D. melanogaster*	*D. simulans*
Full CDS	7	4	2	25	*P* value^a^	7.38E-04***	0.121	2.26E-03**
					α^b^	0.954	0.867	1.000
					DoS^c^	0.562	0.413	0.950
								
N-terminal	2	1	0	1	*P* value^a^	1.000	1.000	1.000
					α^b^	1.000	1.000	NA^d^
					DoS^c^	0.667	0.667	NA^d^
								
S/TP	0	0	0	2	*P* value^a^	1.000	1.000	1.000
					α^b^	NA^d^	NA^d^	NA^d^
					DoS^c^	NA^d^	NA^d^	NA^d^
								
Central	5	2	1	8	*P* value^a^	0.035*	1.000	0.033*
					α^b^	0.950	1.000	1.000
					DoS^c^	0.603	0.600	0.857
								
SPR	0	0	0	3	*P* value^a^	1.000	1.000	1.000
					α^b^	NA^d^	NA^d^	NA^d^
					DoS^c^	NA^d^	NA^d^	NA^d^
								
SAM domain	0	1	1	11	*P* value^a^	1.000	1.000	1.000
					α^b^	NA^d^	NA^d^	NA^d^
					DoS^c^	−0.083	−0.333	NA^d^

Note.—d*N*, number of divergent nonsynonymous substitutions; d*S*, number of divergent synonymous substitutions; p*N*, number of polymorphic nonsynonymous substitutions; p*S*, number of polymorphic synonymous substitutions. Low-frequency variants (<5%) were excluded from analysis. SPR, SAM-proximal region.

a *P* values calculated using Fisher’s exact test, with significant values in italics. ∗, *P* < 0.05. ∗∗, *P* < 0.01. ∗∗∗, *P* < 0.001.

b Proportion of adaptive substitutions (Smith and Eyre-Walker 2002).

c Direction of selection (Stoletzki and Eyre-Walker 2011).

d Uncalculatable, as there are no divergent substitutions.

As was demonstrated previously (Anderson et al. 2009), we found evidence of adaptive selection in *mtrm* sequence evolution, as the MK test for the full-length MSA was significant (*P *<* *0.001), and the DoS was positive (table 2). When we examined the different regions of *mtrm* separately, it was only *mtrm’*s central region that presented a significant MK test result (*P *=* *0.035) as well as a positive value for the DoS (table 2). We also performed polarized MK tests using *mtrm* from *D. yakuba* as an additional outgroup, polarizing fixations on either *D. melanogaster* or *D. simulans*, allowing us to determine whether positive selection occurred in either one or both lineages. For the full *mtrm* sequence, as well as for the central region only, polarized MK tests were significant only when polarizing on *D. simulans* (*P *=* *0.00226 and 0.033, respectively) (table 2 and supplementary table S3, Supplementary Material online), suggesting that the evidence of positive selection in *mtrm* is due to changes occurring in the *D. simulans* lineage.

Taken together, these data suggest that, within *mtrm*, different regions of the gene are under different selective pressures. The MK test and BUSTED results jointly indicate that the high divergence of *mtrm’*s central region is likely due to adaptive selection. Moreover, the aBSREL test suggests that episodic diversifying selection has occurred in *mtrm* on multiple branches within the *melanogaster* group. Interestingly, the PAML analyses performed suggest that *mtrm*—particularly in the well-conserved S/TP region—is primarily experiencing purifying selection. We wondered, then, about the functional consequences of these potentially differing pressures on *mtrm* evolution and sought to explore their effects on both the highly conserved and highly divergent regions of the gene.

### Mtrm’s Best-Conserved Region, the S/TP Region, Contains Eight Critical Residues Required for Its Meiotic Function

The S/TP region of Mtrm, which spans residues V36 to I54 in *D. melanogaster*, is the best-conserved region of the protein (fig. 1*B*). We were unable to calculate a DoS value for the S/TP region of *mtrm* because it contains no nonsynonymous changes, both between and among the *D. melanogaster* and *D. simulans* lines. Additionally, applying fixed-site models to *mtrm* sequences from the *melanogaster* group gave an ω value of 0.010 for the S/TP region, suggesting that it is under strong purifying selection (tables 1 and 2). Previous work demonstrated that the phosphorylated T40, S48, and S52 amino acids, which are fully conserved from *D. melanogaster* to *D. grimshawi*, are critical for the Mtrm::Polo interaction, as mutant Mtrm proteins bearing alanine point mutations of those individual residues were unable to bind Polo or rescue the phenotypes seen in *mtrm* mutant backgrounds (Bonner et al. 2013). However, S39, which is also fully conserved across all Mtrm homologs of the 12 sequenced *Drosophila* species, appears to be dispensable for Mtrm’s interaction with Polo, as expression of a *mtrm^S39A^* mutant transgene behaved like a transgene expressing a wild-type version of Mtrm (Bonner et al. 2013). These data indicate that while Mtrm’s S/TP region is highly conserved, not all its conserved residues are required for function. Therefore, we sought to investigate the role of the other residues contained therein.

To do this, we created multiple FLAG-tagged overexpression transgenic constructs, using *phiC31* site-specific integration (Groth et al. 2004; Venken and Bellen 2012), to perform an alanine-scanning mutagenesis of each residue within the S/TP region. To genetically examine the functionality of the S/TP region point mutants in vivo, we expressed each construct in the *D. melanogaster* female germline using the *nanosGAL4:VP16* driver, denoted *nanosGAL4* (Van Doren et al. 1998). We then tested each of these transgenic constructs for their ability to rescue the meiotic chromosome segregation defect observed in *mtrm/+* heterozygous females. Because the chromosome missegregation observed in *mtrm/+* heterozygotes is limited to affecting segregation of achiasmate chromosomes, these females were also heterozygous for an *X* chromosome balancer (*FM7*), which suppresses exchange between itself and a normal-sequence *X* chromosome. As we have previously shown that the *nanosGAL4* driver system provides adequate Mtrm protein expression for meiotic rescue (Bonner et al. 2013), we recombined the *nanosGAL4* driver onto a third chromosome carrying a deficiency that deletes *mtrm*, Df(3L)66C-T2-10 (Harris et al. 2003), hereafter referred to as *nanosGAL4 mtrm^Df^*.

**Fig. 3.**
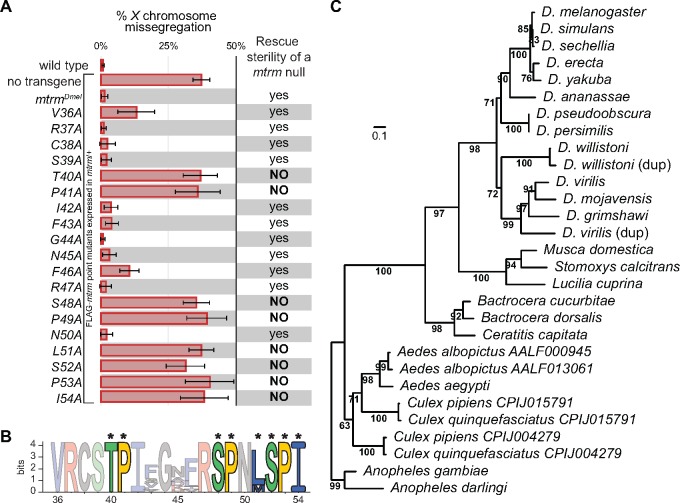
Mtrm’s conserved regions allow for identification of non-*Drosophila* homologs. (*A*) An alanine-scanning mutagenesis of the S/TP region of Mtrm revealed eight residues critical for meiotic function, as assayed by their ability to rescue the achiasmate *X* chromosome missegregation and sterility phenotypes seen in *mtrm*/+ heterozygous and *mtrm* null backgrounds, respectively. (*B*) Sequence logo comparison of S/TP region in *Drosophila* Mtrm homologs. Asterisks mark critical residues. (*C*) Phylogenetic tree of dipteran *mtrm* sequences constructed using IQ-TREE. Scale bar shows number of nucleotide substitutions per site. Branch labels are bootstrap support values, as calculated by UFBoot.

*FM7/X*; *nanosGAL4*/+ females exhibited wild-type *X* chromosome missegregation levels of 0.9%, while *FM7*/*X nanosGAL4 mtrm^Df^*/+ females showed 35.3% *X* chromosome missegregation. When *nanosGAL4 mtrm^Df^* drives expression of a wild-type copy of *mtrm*, denoted *mtrm^Dmel^*, those levels were reduced to 2.6% for the *X* chromosome. This rescue was not seen with expression of *mtrm^T40A^*, *mtrm^S48A^*, or *mtrm^S52A^*, which had *X* chromosome missegregation rates of 37.4%, 35.9%, and 31.7%, respectively (fig. 3*A*), consistent with what we observed previously (Bonner et al. 2013). When assaying across the entire S/TP region, we found five additional amino acids of interest. The Mtrm^P41A^, Mtrm^P49A^, Mtrm^L51A^, Mtrm^P53A^, and Mtrm^I54A^ mutant proteins were also incapable of rescuing the achiasmate chromosome missegregation defect caused by heterozygosity for *mtrm* (fig. 3*A*).

We also assayed the ability of the individual S/TP region point mutants to rescue the sterility phenotype observed in *mtrm* null females. We have previously demonstrated that expression of *mtrm^Dmel^*is able to rescue sterility when expressed in a *mtrm* null background, while expression of *mtrm^T40A^*, *mtrm^S48A^*, or *mtrm^S52A^* cannot (Bonner et al. 2013). Consistent with their inability to rescue chromosome missegregation in *mtrm/+* heterozygous females, expression of *mtrm^P41A^*, *mtrm^P49A^*, *mtrm^L51A^*, *mtrm^P53A^*, or *mtrm^I54A^* in *mtrm* null females was also unable to rescue sterility (fig. 3*A*). Therefore, the highly conserved S/TP region contains eight residues that are critical for Mtrm’s meiotic functions.

### The S/TP Region’s Critical Residues Can Be Used as a Motif to Identify Additional Dipteran Mtrm Homologs

Because of the low level of conservation of *mtrm* sequences even among different *Drosophila* species, we had previously been unable to identify potential homologs outside of the *Drosophila* genus. We wondered, however, whether we could use the critical residues from Mtrm’s S/TP region, represented in figure 3*B*, to search for additional homologs. With Mtrm^Dmel^ as the initial query sequence, we used PHI-BLAST (Pattern Hit Initiated BLAST) (Zhang et al. 1998) coupled with PSI-BLAST (Position-Specific Iterated BLAST) (Altschul et al. 1997) to search the nonredundant protein sequence database from NCBI. Along with returning Mtrm sequence from multiple *Drosophila* species, this search returned protein sequences from numerous non-*Drosophila* dipterans, including sequences from the *Anopheles* genus, which is estimated to have diverged from *Drosophila* ∼260 Ma (Gaunt and Miles 2002). The reciprocal search, using the same PHI-BLAST pattern but with the potential Mtrm homolog from *Anopheles gambiae* as the initial query sequence, returned Mtrm^Dmel^ among its top hits (supplementary table S4, Supplementary Material online). Interestingly, in addition to the two previously described duplications of *mtrm* found in *D. willistoni* and *D. virilis* (Reis et al. 2011), we found paralogous sequences in *Aedes albopictus* and within the *Culex* genus (fig. 3*C*).

Besides containing the conserved S/TP region, all potential homologs contain a SAM domain. However, the sequence identity between Mtrm^Dmel^ and Mtrm from various species of mosquitoes is quite low, at just above 20% for comparisons to either *A. gambiae* or *Aedes aegypti*, which cannot be aligned to *Drosophila* Mtrm with high confidence. Additionally, protein lengths of Mtrm homologs from dipterans are highly variable, ranging from 191 amino acids in *D. mojavensis* to 314 amino acids in *Ceratitis capitata*, a difference of more than 50%. Nearly all this variation falls within the central region, between the S/TP region and the SAM domain of the proteins*.* Despite that high level of sequence variation, a maximum-likelihood phylogenetic tree calculated using all *mtrm* sequences recapitulates published dipteran phylogeny (Wiegmann et al. 2011; Jimenez-Guri et al. 2013) (fig. 3*C*).

To further support these proteins being homologous to *Drosophila* Mtrm, we then looked at the expression of the corresponding genes, for any in which expression data comparing males and females was available, and compared it with *mtrm* expression in *D. melanogaster*, which is expressed at a high level but is female-specific (Chintapalli et al. 2007). All genes with available expression data (supplementary table S5, Supplementary Material online) showed similar patterns (Gnad and Parsch 2006; Marinotti et al. 2006; Koutsos et al. 2007; Dissanayake et al. 2010; Baker et al. 2011; Chen et al. 2015; Meisel et al. 2015), indicating that while overall *mtrm* sequence is highly divergent, its expression pattern, like the S/TP region, is highly conserved across the dipteran order.

### Distant *Drosophila* Mtrm Homologs Can Fulfill the Roles of Mtrm in Meiosis

Because only the S/TP region and SAM domain of Mtrm are conserved across the dipteran order, we wondered whether it is only *mtrm’*s conserved regions that are required for Mtrm protein function. If so, the rest of the protein might be less critical, and its high level of divergence could be due to relaxed selective constraint, where nonsynonymous mutations arising in those unconserved regions would be selectively neutral. If this is the case, one might expect even the most divergent *Drosophila mtrm* homolog to be fully functional in *D. melanogaster*. Conversely, *mtrm’*s divergence could be driven by positive selection, where sequence changes lead to adaptive advantages, in which case more divergent *mtrm* homologs might show some level of impairment in their function when expressed in the *D. melanogaster* female germline. We therefore sought to investigate the effects of expressing divergent forms of the Mtrm protein during *D. melanogaster* female meiosis.

To do this, we created FLAG-tagged overexpression transgenic constructs for *mtrm* homologs found in those *Drosophila* species sequenced by the Drosophila 12 Genomes Consortium (2007). Specifically, we examined *mtrm* homologs from *D. simulans* (*mtrm^Dsim^*), *D. sechellia* (*mtrm^Dsec^*), *D. erecta* (*mtrm^Dere^*), *D. yakuba* (*mtrm^Dyak^*)*, D. ananassae* (*mtrm^Dana^*), *D. pseudoobscura* (*mtrm^Dpse^*), *D. willistoni* (*mtrm^Dwil^*), *D. virilis* (*mtrm^Dvir^*), *D. mojavensis* (*mtrm^Dmoj^*), and *D. grimshawi* (*mtrm^Dgri^*). The nucleotide sequence for *mtrm* in *D. persimilis* is identical to *mtrm^Dpse^*, so a single transgenic construct was created to represent both. In addition, because it is known that codon usage in *D. willistoni* genes differs greatly from other *Drosophila* species (Powell et al. 2003), we optimized the coding sequence for *mtrm^Dwil^* based on *D. melanogaster* codon usage. We did the same for *mtrm^Dgri^*, as the nonoptimized construct showed reduced expression compared with the other constructs. We expressed each construct in the *D. melanogaster* female germline to assess their ability to express the appropriate protein products by western blotting (supplementary fig. S2, Supplementary Material online). As all constructs are expressed at a comparable level, we then tested for their ability to rescue the chromosome missegregation and sterility defects seen in *mtrm*/+ heterozygous and *mtrm* null females, respectively.

**Fig. 4.**
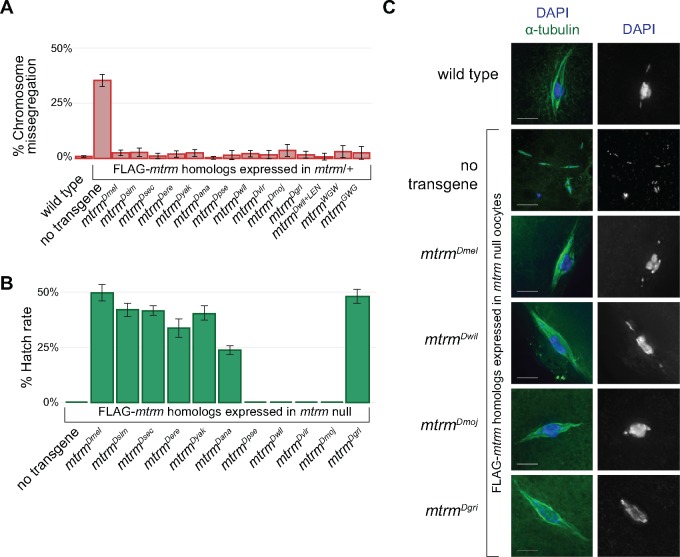
Functionality of Mtrm homologs in *Drosophila melanogaster* female meiosis. (*A*) While levels of achiasmate *X* chromosome missegregation rates were high in *mtrm*/+ heterozygotes, expression of all *mtrm* homologs was able to rescue the missegregation to wild-type levels. (*B*) Graph of hatch rates when *mtrm* homologs were expressed in a *mtrm* null background. (*C*) Late-stage oocytes at prometaphase I, stained with DAPI (blue) and α-tublin (green). Scale, 5 μm. Wild-type oocytes showed the expected karyosome morphology, with the chromosome mass centered on a tapered, bipolar spindle. In *mtrm* null oocytes, karyosomes were abnormal, often with multiple separated chromosome masses on multiple spindles, indicative of meiotic catastrophe. Expression of *mtrm^Dmel^*, *mtrm^Dwil^*, *mtrm^Dmoj^*, or *mtrm^Dgri^* in *mtrm* null oocytes rescued the meiotic catastrophe phenotype.

As before, we expressed each of the *mtrm* homolog transgenic constructs in *FM7*/*X*; *nanosGAL4 mtrm^Df^*/+ females and assayed their ability to rescue *X* chromosome missegregation. Expression of any of the *mtrm* homolog transgenic constructs in *mtrm*/*+* heterozygous females had *X* chromosome missegregation levels comparable to what was observed with expression of *mtrm^Dmel^* (fig. 4*A*). These data indicate that all the *mtrm* homologs are capable of rescuing the haploinsufficient meiosis I chromosome missegregation phenotype of *mtrm* in *D. melanogaster*.

We next assayed the ability of the *mtrm* homologs to rescue the sterility phenotype seen in *nanosGAL4 mtrm^Df^/mtrm^126^*, or *mtrm* null, females. While embryos laid by *mtrm* null females never hatched, embryos laid by *mtrm^Dmel^*/+; *nanosGAL4 mtrm^Df^/mtrm^126^* females had a hatch rate of ∼50% (fig. 4*B*), consistent with previous results (Bonner et al. 2013). Rescue of fertility was also seen with expression of *mtrm^Dsim^*, *mtrm^Dsec^*, *mtrm^Dere^*, *mtrm^Dyak^, mtrm^Dana^*, and *mtrm^Dgri^*; however, *nanosGAL4 mtrm^Df^/mtrm^126^* females expressing either *mtrm^Dpse^*, *mtrm^Dwil^*, *mtrm^Dvir^*, or *mtrm^Dmoj^* remained completely sterile (fig. 4*B*). Going forward, these four *mtrm* homologs will be denoted as the distant *mtrm* homologs, as all are from *Drosophila* species outside of the *melanogaster* group, and all diverged from *D. melanogaster* over 50 Ma (Tamura et al. 2004). Interestingly, expression of *mtrm^Dgri^*, the most divergent homolog, was able to rescue the sterility in *mtrm* null females to a similar level as *mtrm^Dmel^* and will be discussed below.

To understand the inability of the distant *mtrm* homologs to rescue sterility, we compared prometaphase I oocytes from *nanosGAL4 mtrm^Df^/mtrm^126^* females to wild-type oocytes as well as to oocytes from *nanosGAL4 mtrm^Df^/mtrm^126^* females expressing one of the *mtrm* homolog constructs. As we previously demonstrated (Bonner et al. 2013), at prometaphase I, *nanosGAL4 mtrm^Df^/mtrm^126^* oocytes exhibited meiotic catastrophe, with nuclei that were highly fragmented, often arranged on multiple spindles. Comparable to wild-type oocytes, *nanosGAL4 mtrm^Df^/mtrm^126^* oocytes expressing *mtrm^Dmel^* had intact nuclei centered on a tapered, bipolar spindle, often with the small, dot-like fourth chromosomes separated from the main chromosome mass, indicative of prometaphase I (fig. 4*C*). Interestingly, nuclei from *mtrm* null oocytes expressing *mtrm^Dwil^* or *mtrm^Dmoj^*, representative of the distant Mtrm homologs, as well as *mtrm^Dgri^*, were also comparable to wild type at prometaphase I (fig. 4*C*). These results strongly suggest that even the most distant *mtrm* homologs are competent to rescue both assayed meiotic defects—chromosome missegregation and meiotic catastrophe—observed when *mtrm* mutants are made hetero- or homozygous, respectively, in *D. melanogaster*.

### The Inability of the Distant Mtrm Homologs to Rescue Sterility in an *mtrm* Null Background Is Due to Defects in Early Embryonic Mitoses, Not an Inability to Interact with Polo

**Fig. 5.**
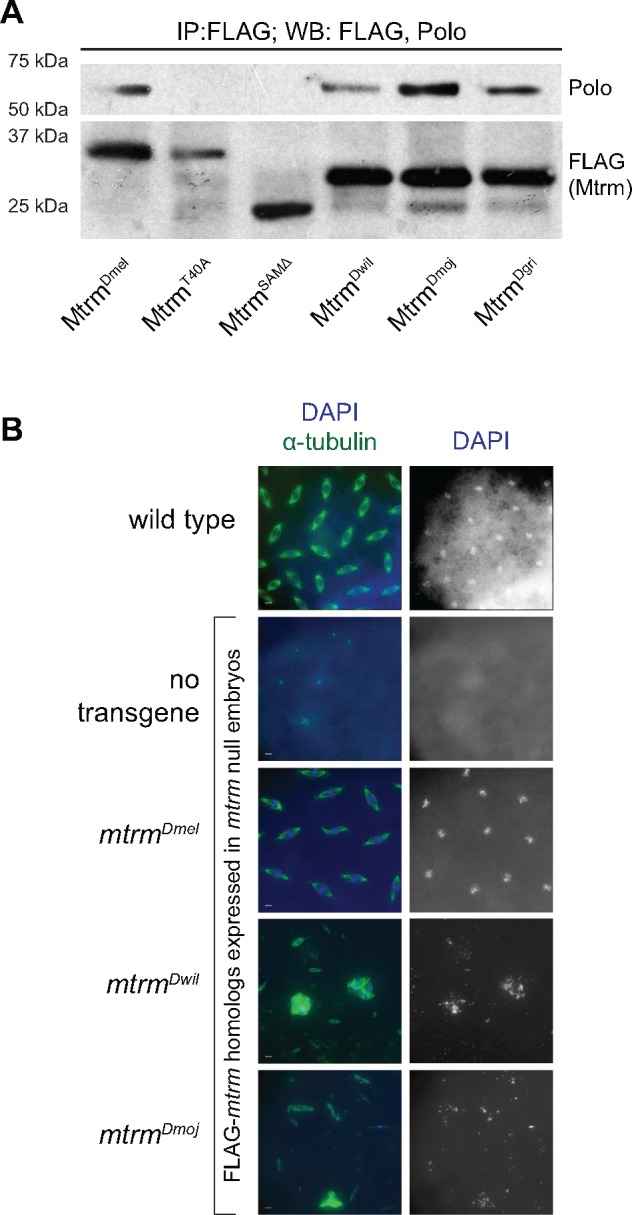
Expression of distant *mtrm* homologs caused defects in embryos despite their ability to bind Polo. (*A*) Co-IP of Polo by FLAG-Mtrm homologs from late-stage oocytes showed that Mtrm^Dmel^, Mtrm^Dwil^, Mtrm^Dmoj^, and Mtrm^Dgri^ were all able to bind Polo, while negative controls Mtrm^T40A^ and Mtrm^SAMΔ^ did not. (*B*) Embryos aged 0–2 h, laid by females expressing one of the *mtrm* homolog constructs in a *mtrm* null background. Embryos were stained with DAPI (blue) and α-tublin (green). Scale, 5 μm. The majority of embryos expressing *mtrm^Dmel^* in this background were comparable to wild type, while expression of either *mtrm^Dwil^* or *mtrm^Dmoj^* resulted in mitotic defects.

To understand the inability of the distant Mtrm homologs to rescue sterility, we first examined whether they were able to physically interact with Polo, which is a critical role for Mtrm. We immunoprecipitated FLAG-tagged Mtrm homolog proteins from ovaries, followed by western blotting with antibodies that recognize FLAG and Polo. Consistent with previous results (Bonner et al. 2013), Mtrm^Dmel^ was able to pull down Polo, while neither Mtrm^T40A^, which contains a point mutation of a critical residue in the S/TP region, nor Mtrm^SAMΔ^, a Mtrm construct lacking its C-terminal SAM domain, was able to interact with Polo by co-immunoprecipitation (co-IP) (fig. 5*A*). As with Mtrm^Dmel^, the Mtrm^Dwil^, Mtrm^Dmoj^, and Mtrm^Dgri^ proteins were also able to interact with Polo (fig. 5*A*). Thus, these data suggest that the inability of the distant Mtrm homologs to rescue sterility in a *mtrm* null background is not due to defects that occur in meiosis, as they are able to interact with Polo in the ovary. Instead, we hypothesized that the defects caused by the distant Mtrm homologs occur postmeiotically in the early syncytial embryo, where they may continue to inhibit Polo.

When we examined embryos from *mtrm* null mothers, the majority were empty and lacked identifiable nuclei or spindles, which is consistent with previous findings (Bonner et al 2013). For those in which chromatin could be identified, that chromatin was highly fragmented and only rarely associated with small, anastral spindles. Together, these observations indicate that there is a failure to complete meiosis in *mtrm* null embryos (fig. 5*B*). In contrast, more than half of embryos laid by *mtrm* null females expressing *mtrm^Dmel^* showed normal mitotic development (fig. 5*B*). When either *mtrm^Dwil^* or *mtrm^Dmoj^* was expressed in a *mtrm* null background, the resulting embryos did not show normal mitotic development, but the defects we saw were not the same as those observed in embryos laid by *mtrm* null mothers. Instead we often observed various mitotic defects, including large masses of fragmented chromatin that were usually associated with aberrant astral spindles, suggesting that the oocytes completed meiosis and attempted the earliest mitotic divisions (fig. 5*B*).

These phenotypes are not unlike those seen in *polo* mutant embryos, which are able to undergo early mitotic cycles but have highly disorganized, polyploid nuclei (Sunkel and Glover 1988). These defects are also similar to those occasionally seen in embryos laid by females overexpressing *mtrm^Dmel^* with the maternal alpha-tubulin *GAL4* driver (Whitfield et al. 2013). In that study, the authors demonstrated that developmental defects were caused by excess Mtrm in the early embryo, as the postmeiotic elimination of Mtrm is critical for proper embryonic development (Whitfield et al. 2013). Additionally, as Mtrm is known to interact with Polo in an inhibitory manner (Xiang et al. 2007), Whitfield et al. (2013) posited that those observed embryonic defects were due to prolonged inhibition of Polo. The defects they observed were exacerbated when *mtrm^Dmel^* was overexpressed in a *polo/+* heterozygous background, yet overexpression of *mtrm^T40A^* in a *polo/+* heterozygous background resulted in embryos with no such defects (Whitfield et al. 2013). Therefore, we wondered whether the ability of distant Mtrm homologs to interact with Polo extended beyond the meiosis-to-mitosis transition, thus reducing the amount of active Polo available in the early embryonic divisions.

### Expression of Distant *mtrm* Homologs in Wild-Type Females Results in a Dominant-Negative Phenotype in Embryos

**Fig. 6.**
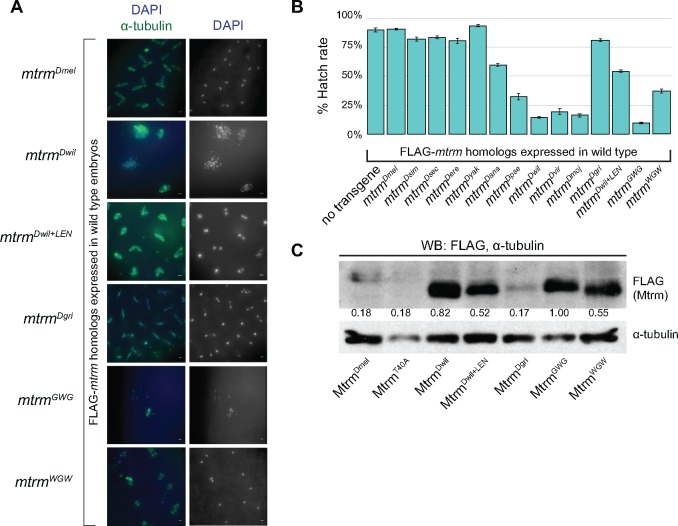
Dominant-negative phenotypes caused by expression of distant *mtrm* homologs. (*A*) Early embryos, age 0–2 h, laid by females expressing one of the *mtrm* homolog constructs in an otherwise wild-type background. Embryos were stained with DAPI (blue) and α-tublin (green). Scale, 5 μm. Expression of either *mtrm^Dmel^* or *mtrm^Dgri^* resulted in wild-type-like embryos, while multiple mitotic defects could be seen with expression of *mtrm^Dwil^*, *mtrm^GWG^*, or *mtrm^WGW^*, and to a lesser extent with expression of *mtrm^Dwil+LEN^*. (*B*) Graph of hatch rates when *mtrm* homologs were expressed in a wild-type background. (*C*) Western blot of Mtrm protein levels in early, 0- to 2-h-old embryos. Numbers below FLAG bands are relative amounts of FLAG-tagged protein, normalized to α-tublin levels.

To determine whether the distant *mtrm* homologs are inactivating Polo in the early embryonic divisions, we next expressed them in wild-type females (i.e., those that possess two endogenous, wild-type copies of the *mtrm* gene). Females that were expressing *mtrm^Dmel^* in their germline produced embryos that were similar to embryos from wild-type mothers (fig. 6*A*). The same was not true, however, for females expressing *mtrm^Dwil^*. Instead, expression of *mtrm^Dwil^* in wild-type females often resulted in embryos that had multiple developmental defects (fig. 6*A*), similar to those seen in embryos from *mtrm* null mothers expressing *mtrm^Dwil^* (fig. 5*B*). This dominant-negative effect suggests that Mtrm^Dwil^ is binding Polo beyond meiosis and disrupting the early embryonic mitoses.

Not surprisingly, expression of the distant *mtrm* homologs that were unable to rescue sterility in *mtrm* null females also had a dominant-negative effect on hatch rate when expressed in wild-type females. Embryos laid by *nanosGAL4*/+ females had a hatch rate of 90.1%, and embryos from *nanosGAL4*/+ females expressing *mtrm^Dmel^* had a comparable hatch rate of 91.4%. Similar rates were seen when *mtrm^Dsim^*, *mtrm^Dsec^*, *mtrm^Dere^*, *mtrm^Dyak^*, or *mtrm^Dgri^* were expressed. Expression of *mtrm^Dana^* resulted in a moderate reduction in hatch rate to 59.2%, but expression of *mtrm^Dpse^*, *mtrm^Dwil^*, *mtrm^Dvir^*, or *mtrm^Dmoj^* led to severely reduced hatch rates of 32.5%, 14.7%, 19.4%, and 16.4%, respectively (fig. 6*B*). Therefore, expression of the distant *mtrm* homologs has a dominant-negative effect on early embryonic development. As this is likely due to the distant Mtrm homologs’ continued ability to inhibit Polo in the early embryo, we hypothesized that expressing each of the *mtrm* homologs in *polo*/*+* heterozygous females should result in even greater defective phenotypes, similar to what was previously observed by Whitfield *et al* (2013). Indeed, we observed a significant reduction in hatch rate upon expression of all the individual Mtrm homologs in *polo/+* heterozygotes compared with their expression in a wild-type background (supplementary fig. S3, Supplementary Material online).

### An APC/C Recognition Motif That Is Lacking in Distant Mtrm Homologs Can Partially Suppress Their Dominant-Negative Effects and Increase Their Ability to Be Properly Degraded

In order for the distant Mtrm homologs to continue to inhibit Polo in early embryos, they must persist beyond the oocyte-to-embryo transition, the time when Mtrm^Dmel^ has been demonstrated to be drastically downregulated (Arbeitman et al. 2002; Whitfield et al. 2013). A previously characterized LxExxxN (denoted LEN) APC/C recognition motif in the N-terminal region of Mtrm has been shown to aid in the protein’s proper degradation (Whitfield et al. 2013). However, the LEN degron motif is conserved only within the *melanogaster* group and therefore is not found in any of the distant Mtrm homologs (supplementary fig. S1, Supplementary Material online). We hypothesized that the dominant-negative effects caused by expression of the distant *mtrm* homologs in *D. melanogaster* might be due to an inability of those proteins to be properly degraded prior to the syncytial embryonic divisions. To test this, we created a transgenic overexpression construct for Mtrm^Dwil^ in which we added the LEN degron motif (denoted Mtrm^Dwil+LEN^) (supplementary fig. S4, Supplementary Material online).

Protein expression with the Mtrm^Dwil+LEN^ was comparable in late-stage oocytes to protein levels seen with the other Mtrm homologs (supplementary fig. S2, Supplementary Material online). Also, similar to the other Mtrm homolog transgenes, expression of *mtrm^Dwil+LEN^* is able to rescue the chromosome missegregation defect in *FM7*/*X; nanosGAL4 mtrm^Df^*/+ females (fig. 4*A*). However, the hatch rate of embryos laid by *nanosGAL4*/+ females expressing *mtrm^Dwil+LEN^* was 59.0%, intermediate to the hatch rates seen with *mtrm^Dmel^* and *mtrm^Dwil^* (fig. 6*B*). The development phenotypes in those early embryos was also intermediate (fig. 6*A*), with 15/31 that were comparable to wild type, and only 1/31 showing the fragmented chromatin masses we observed with *mtrm^Dwil^*. Instead, the most prominent aberrant phenotypes we observed in *mtrm^Dwil+LEN^* embryos were nuclei with unattached centrosomes (7/31). Interestingly, this phenotype is similar to what is seen in embryos with reduced *polo* or in embryos treated with a chemical inhibitor of Polo-like kinase 1, BI2536 (Wang et al. 2011), supporting the idea that expression of the distant Mtrm homologs leads to ectopic inhibition of Polo in the early embryo.

We then looked at protein levels of the different Mtrm homologs present in the early embryo to confirm their continued presence at a time when endogenous Mtrm has been degraded. While the expected reduction of Mtrm protein between late-stage oocytes and embryos was observed with Mtrm^Dmel^, protein levels remained high in embryos for Mtrm^Dwil^ (fig. 6*C* and supplementary fig. S2, Supplementary Material online). Addition of the LEN degron motif to Mtrm^Dwil^ allowed for increased degradation of the protein (as seen in the lower amount of protein when normalized to α-tubulin) in Mtrm^Dwil+LEN^ embryos compared with Mtrm^Dwil^ (fig. 6*C*). Taken together, these data support the hypothesis that the dominant-negative effects caused by expression of the distant Mtrm homologs are due to their inability to be properly degraded, and that degradation is at least partially aided by the LEN degron.

### Expression of Chimeric Constructs Demonstrates That Mtrm’s Central Region Affects Its Function and/or Degradation

While the LEN degron motif aids in the degradation of Mtrm protein, there must be other residues required, as its addition to Mtrm^Dwil^ cannot fully rescue its dominant-negative phenotypes. Also, a previous study has shown that Mtrm^L21A^, which contains a point mutation in the critical leucine residue of the LEN degron motif, is able to be partially degraded in embryos, suggesting that the LEN degron motif is necessary but not sufficient for full Mtrm protein degradation (Whitfield et al. 2013). Additionally, Mtrm^Dgri^, the most distantly related Mtrm^Dmel^ homolog for which we created a transgenic construct, functions similarly to Mtrm^Dmel^, despite the fact that Mtrm^Dgri^ does not contain the LEN degron motif. As with all of the Mtrm homologs, expression of *mtrm^Dgri^*rescues the chromosome missegregation defects in a *mtrm* heterozygous background, and Mtrm^Dgri^ is able to interact with Polo when assayed by co-IP (figs. 4*A* and 5*A*). However, unlike the other distant Mtrm homologs, expression of *mtrm^Dgri^*rescues sterility in a *mtrm* null background and does not result in a dominant-negative phenotype when expressed in wild-type females (figs. 4*B*, 6*A*, and 6*B*). Also, Mtrm^Dgri^ protein levels appear to be reduced in the embryo to the same level as Mtrm^Dmel^, despite the fact that Mtrm^Dgri^ does not contain the LEN degron motif (fig. 6*C* and supplementary fig. S1, Supplementary Material online).

The presence or absence of the LEN degron motif accounts for much of the variation found within Mtrm’s N-terminal region, but sequence comparison of the unconserved central region among all Mtrm homologs is difficult, as it is so divergent that MSA algorithms cannot confidently align it (supplementary fig. S1, Supplementary Material online). Therefore, we wanted to explore whether the central region of the Mtrm protein might contain residues or motifs that are also critical for protein degradation or stability. If so, we wondered whether those differences might account for the ability of Mtrm^Dgri^ to function more similarly to Mtrm^Dmel^ than to the distant Mtrm homologs.

To investigate this, we created two additional transgenes that were chimeric constructs for Mtrm^Dwil^ and Mtrm^Dgri^. For these we swapped out their central regions, such that Mtrm^Dwil^ with the central region of Mtrm^Dgri^ is denoted Mtrm^WGW^, and Mtrm^Dgri^ with the central region of Mtrm^Dwil^ is denoted Mtrm^GWG^ (supplementary fig. S4, Supplementary Material online). We hypothesized that if the central region of Mtrm^Dgri^ contained residues or motifs that were affecting its degradation or stability, its addition to Mtrm^Dwil^ in the Mtrm^WGW^ construct should result in an amelioration of the dominant-negative phenotype that we observe with Mtrm^Dwil^ alone. At the same time, expression of the Mtrm^GWG^ construct should result in more severe defects than are seen with Mtrm^Dgri^ alone. Conversely, if the central region of Mtrm^Dgri^ is not required for degradation or stability, we would not expect to see an effect upon its replacement in either chimeric construct.

As with *mtrm^Dwil^* and *mtrm^Dgri^*, expression of either *mtrm^WGW^* or *mtrm^GWG^* can rescue the chromosome missegregation found in *mtrm* heterozygotes—evidence of their functionality during meiosis (fig. 4*A*). However, expression of either construct led to embryos that were developmentally abnormal the majority of the time, though the phenotypes were much worse in *mtrm^GWG^* (fig. 6*A*). Consistent with those results, embryos laid by mothers expressing either chimeric construct hatched at a reduced level compared with expression of *mtrm^Dmel^*. Embryos expressing *mtrm^WGW^* had a hatch rate of 37.0%, which is greater than the hatch rate of 14.7% seen with expression of *mtrm^Dwil^* but is well below the rate of 81.2% that was seen with *mtrm^Dgri^* (fig. 6*B*). Protein levels in embryos laid by mothers expressing *mtrm^WGW^* were also intermediate to those seen in *mtrm^Dwil^* and *mtrm^Dgri^* (fig. 6*C*). Females expressing *mtrm^GWG^*, however, were nearly sterile, as their embryos had a hatch rate of 9.6% (fig. 6*B*), and protein levels of Mtrm^GWG^ remained quite high in embryos (fig. 6*C*). Taken together, these data suggest that the central region of Mtrm^Dgri^ is critical, as its replacement leads to near sterility. Also, its addition to Mtrm^Dwil^ increases the viability of those embryos, though as was the case with Mtrm^Dwil+LEN^, Mtrm^WGW^ does not fully rescue the dominant-negative phenotypes we see with Mtrm^Dwil^.

## Discussion

In *Drosophila*, as in many organisms, strong tissue bias in a gene’s expression is positively correlated with its evolutionary rate (Larracuente et al. 2008). Indeed, multiple studies have shown that proteins involved in reproduction, particularly those with strong sex-biased expression, evolve rapidly (Swanson and Vacquier 2002; Jagadeeshan and Singh 2005; Haerty et al. 2007). Conversely, essential genes and/or those that are highly expressed tend to show higher levels of conservation (Larracuente et al. 2008). Here, we have studied the molecular evolution, and the functional consequences thereof, of the *mtrm* gene. Some regions of *mtrm* are highly conserved, as might be expected of a gene that is highly expressed and is critical for female fertility in *D. melanogaster*. Other regions are rapidly diverging, consistent with its strong tissue-biased expression, which is limited to the ovary.

Our analysis has shown that *mtrm* homologs that diverged from *D. melanogaster* over 60 Ma are able to rescue meiotic phenotypes when expressed in *D. melanogaster mtrm* mutant females. However, expression of many of the more divergent homologs results in a dominant-negative embryonic lethality in *D. melanogaster*, due to an inability of their protein products to be properly degraded in the early embryo. Consistent with previous data (Whitfield et al. 2013), we have shown that the timely degradation of Mtrm protein is at least partially due to the presence of a previously described LEN degron found near Mtrm’s N-terminus.

Additionally, we have demonstrated that Mtrm’s small but highly conserved S/TP region contains multiple residues that are critical for the Mtrm::Polo interaction. Using those critical residues as a motif for BLAST searches, we were able to find potential non-*Drosophila* homologs in other dipteran species (supplementary table S4, Supplementary Material online). For those species with available expression data, the genes we identified as being homologous to *mtrm^Dmel^* also show strongly female-biased expression patterns (supplementary table S5, Supplementary Material online). Sequence conservation among the dipteran *mtrm* homologs is quite low, however, and we were unable to identify potential homologs from any non-dipteran insects. Interestingly, along with the two previously identified independent duplications of *mtrm* that have occurred in *Drosophila* (Reis et al. 2011), we found evidence of two additional independent duplications in mosquitoes (fig. 2*C*).

Taken together, these results suggest that there are multiple selective pressures driving *mtrm* evolution. Changes in either the S/TP region or the SAM domain would appear to be highly deleterious, as those regions are under strong purifying selection. As it is only the conserved regions that are critical for Mtrm’s meiotic function, one might expect the high levels of divergence within *mtrm’*s unconserved regions to be due to relaxed constraint, with both synonymous and nonsynonymous changes being effectively neutral. Surprisingly, that does not seem to be the case. Instead we detected a signature of positive selection when comparing *mtrm* sequences from multiple lines of *D. melanogaster* and *D. simulans* (table 2), which was consistent with findings from a previous study (Anderson et al. 2009). When narrowing down those analyses by gene region, it was only in *mtrm’*s central region that the signature of positive selection was significant. Our analyses have also shown that episodic positive selection has occurred across branches within the *melanogaster* group (fig. 2*A*).

Our functional studies using chimeric *mtrm* homologs, where we swapped the central regions of *mtrm^Dwil^*and *mtrm^Dgri^*, also support the idea of positive selection affecting that region, as we would not expect those chimeric constructs to perform differently from their nonchimeric homolog “parents” if the high divergence of the central region was simply due to relaxed constraint. However, as the central region of *mtrm* is so poorly conserved that it cannot be reliably aligned among the 12 *Drosophila* homologs (supplementary fig. S1, Supplementary Material online), we have not been able to determine whether it is evolving under positive selection across the genus. Therefore, it is possible that the phenotypic differences we see upon expression of the distant *mtrm* homologs are not attributed to adaptive changes that have occurred in the central region.

One hypothesis as to why regions of *mtrm* may be under positive selection relates to the role Mtrm plays in meiotic timing. Mtrm has previously been shown to affect the progression of meiosis, as *mtrm* mutant oocytes precociously break down their nuclear envelopes (Xiang et al. 2007). It has been suggested that changes in meiotic duration can be adaptive, affecting a species’ life cycle in a particular environment (Bennett 1977), and in *Drosophila*, the duration of meiosis may be a trait that is under selection (Reis et al. 2011). Additionally, Polo activity is critical for early embryonic development (Sunkel and Glover 1988), and Mtrm’s interaction with Polo is inhibitory (Xiang et al. 2007). Therefore, it is possible that changes in the timing or efficiency of Mtrm degradation could affect the availability of Polo to function during the early syncytial mitoses, and substitutions in *mtrm* that affect this process could potentially be advantageous in different environments.

## Materials and Methods

### *Drosophila* Stocks

The nanosGAL4:VP16 driver located on chromosome 3 was used to drive expression of transgenic constructs in the female germline (Van Doren et al. 1998). The wild-type *D. melanogaster* controls had the genotype *y w; nanosGAL4:VP16/+; sv^spa-pol^* for all experiments, except those where we were measuring chromosome missegregation (figs. 2*A* and 3*A*). For those experiments, the wild-type genotype was *FM7w/y w; nanosGAL4:VP16/+; sv^spa-pol^*. The *mtrm* mutant alleles used were: *Df(3L)66C-T2-10*, a deficiency that uncovers *mtrm* (Harris et al. 2003), denoted *mtrm^Df^*; and *mtrm^126^*, a null P-element excision allele (Xiang et al. 2007). A recombinant stock carrying both *nanosGAL4:VP16* and *mtrm^Df^* on the third chromosome, referred to as *nanosGAL4 mtrm^Df^*, was used to drive expression in a *mtrm* mutant background. The *mtrm* null background refers to genotype *y w; nanosGAL4:VP16 mtrm^Df^/mtrm^126^; sv^spa-pol^*. The *polo* allele used was *polo^16-1^* (Roseman et al. 1995). The *mtrm^Dmel^*, *mtrm^T40A^*, *mtrm^S48A^*, *mtrm^S52A^*, and *mtrm^SAMΔ^* transgenic stocks were described previously (Bonner et al. 2013). All stocks were maintained at 24 °C under standard conditions.

### Molecular Biology

Sequences for *mtrm^Dwil^* and *mtrm^Dgri^* were codon-optimized using the codon optimization tool available from Integrated DNA Technologies (http://www.idtdna.com/CodonOpt; last accessed October 31, 2018). The chimeric *mtrm^WGW^* and *mtrm^GWG^* constructs were created using codon-optimized sequences. Synthetic gene fragments (supplementary data, Supplementary Material online) were generated by Integrated DNA Technologies for *mtrm^Dwil^*, *mtrm^Dgri^*, *mtrm^WGW^*, and *mtrm^GWG^*, with 5′ *Not*I and 3′ *Bam*HI restriction sites added. For the remaining *Drosophila* species, the *mtrm* coding regions were PCR-amplified from those species, each with 5′ *Not*I and 3′ *Bam*HI restriction sites added. To construct the *mtrm* point mutant transgenes and the *mtrm^Dwil+LEN^* transgene, the *mtrm^Dmel^* or *mtrm^Dwil^* coding regions were subcloned into *pBluescriptSKII*^+^. The Stowers Molecular Biology facility made the point mutations in *mtrm^Dmel^* or inserted the LEN degron motif into *mtrm^Dwil^* using the Quik Change II XL Site-Directed Mutagenesis Kit from Stratagene. All transgenic strains used were then created by subcloning the coding region of *mtrm* into the *pUASp-attB-3XFLAG* vector, as described previously (Bonner et al. 2013).

### Missegregation Assays

For missegregation assays, individual *FM7w/y w; transgene*/*+; nanosGAL4:VP16 mtrm^Df^/+; sv^spa-pol^* females were crossed to *attached-XY, y+ v f B*; *C(4)RM, ci ey^R^* males, and *X* chromosome missegregation levels were measured for at least 200 progeny per genotype, as described by Hawley et al. (1992).

### Hatch Count Assays

To assay hatch rates, *mtrm* transgenes were expressed in the following backgrounds: *nanosGAL4:VP16/+, nanosGAL4:VP16 mtrm^Df^*/*mtrm^126^*, or *nanosGAL4:VP16/polo^16-1^*. Transgene-bearing females were crossed to *y w/y + Y; sv^spa-pol^* males and allowed to lay on grape plates for 2 h. Parents were removed, and grape plates were held at 24 °C for 24 h, after which hatched and unhatched embryos were counted and recorded. To simply measure rescue of sterility, as in figure 2*A*, *y w; transgene/+; nanosGAL4:VP16 mtrm^Df^/mtrm^126^; sv^spa-pol^* females were placed in vials with *y w/y + Y; sv^spa-pol^* males, and rescue of sterility was determined 1 week later by the presence or complete absence of larvae.

### Cytology

Ovaries from 2- to 3-day-old yeasted females were dissected and fixed with cacodylate/formaldehyde as described by Hughes et al. (2011). For embryos, mated females laid on grape plates for 2 h, and then embryos were fixed in heptane/methanol as described by Bonner et al. (2013). Rat anti-α-tublin primary antibody (1:250, BioRad) was used with Alexa-488 or Alexa-555 conjugated secondary antibody (Molecular Probes, 1:350). DNA was then labeled with DAPI (2 μg/ml), and samples were mounted in ProLong Gold (Invitrogen).

For all imaging, the DeltaVision microscopy system (Applied Precision), equipped with an inverted Olympus 1670 microscope and a high-resolution CCD camera, was used. All acquired images were then deconvolved using SoftWoRx software (Applied Precision).

### Co-IPs and Western Blots

Sample preparation for western blotting and co-IP of FLAG-tagged transgenic flies was done as described by Bonner et al. (2013). Primary antibodies used were rat anti-α-tublin (1:100,000, BioRad), mouse anti-FLAG (1:10,000, Sigma), and mouse anti-Polo (1:100, gift from the Claudio Sunkel Laboratory, Portugal). All horseradish peroxidase secondary antibodies were used at 1:10,000. The western blots were developed using SuperSignal West Pico PLUS Chemiluminescent Substrate (ThermoFisher Scientific), and the signal was captured on film.

### Identification of *mtrm* Homologs

The *mtrm* homologs from 12 *Drosophila* species have been described previously (Reis et al. 2011). To obtain *mtrm* sequence from additional *Drosophila* species, *mtrm^Dmel^* was used as a query sequence for BLAST. Sequences from *D. mauritiana*, *D. orena*, *D. santomea*, and *D. teissieri* were obtained from data deposited at NCBI Sequence Read Archive (SRA), under accession numbers SRR6425993, SRR5382770, SRR5860605, and SRR5860571, respectively. As before, *mtrm^Dmel^* was used as a query sequence to BLAST against the SRA data sets, and the top 100 reads were acquired for each species. Those reads were then aligned to *mtrm^Dmel^*sequence to create a consensus sequence for each.

To identify potential non-*Drosophila mtrm* homologs, we used the critical residues from Mtrm’s S/TP region ([ST]-P-X(5, 8)-S-P-X-[LIM]-S-P-I) as the PHI pattern for PHI-BLAST (Zhang et al. 1998), implemented in pBLAST from NCBI, using Mtrm^Dmel^ as the initial query sequence. Those results were then used as a query for PSI-BLAST (Altschul et al. 1997). A reciprocal PHI-BLAST search was performed using Mtrm sequence from *A. gambiae* as the query. BLAST results are available in supplementary table S4, Supplementary Material online.

### MSAs and Phylogenetic Tree Construction

The alignment of *mtrm* sequences was done with the PRANK_+F_ algorithm (Loytynoja and Goldman 2008), implemented in GUIDANCE2, which also assigned a reliability score for each column in the alignment (Sela et al. 2015). The following substitution models were selected by ModelFinder (Kalyaanamoorthy et al. 2017): TIM+F+R3 for the 12 *Drosophila* species MSA, TIM+F + I+G4 for the *melanogaster* group MSA, and TPM3u+F + I+G4 for the dipteran MSA.

Phylogenetic trees were then created by inference of maximum likelihood phylogeny by IQ-TREE (Nguyen et al. 2015) and UFBoot (Minh et al. 2013), using the parameters “-bb 100000 -alrt 100000.” The S/TP region sequence logo was generated with WebLogo 3.6 (Crooks et al. 2004) using amino acid MSAs.

### Analyses for Natural Selection

Codon-based models were run as implemented in the codeml program in PAML (Yang 1997). Results from codeml displayed in table 1 use a 1/61 codon frequency model and an initial ω value of 0.1, though results were consistent using starting ω values ranging from 0.001 to 2, as well as using a codon frequency model of F3x4. The aBSREL (Smith et al. 2015) and BUSTED (Murrell et al. 2015) analyses were performed on the datamonkey server (Delport et al. 2010), with the entire phylogeny set as the foreground.

For the MK test, *D. simulans* sequences were retrieved from http://www.molpopgen.org/markdown/data.html; last accessed October 31, 2018 (Rogers et al. 2014) and the *Drosophila* Population Genomics Project (Begun et al. 2007), as well as by BLASTing *mtrm^Dsim^* sequence against whole-genome shotgun contigs available through NCBI. Sequences for *D. melanogaster* were retrieved from the *Drosophila* Genetics Reference Panel (Mackay et al. 2012). Information for all lines used is available in supplementary table S2, Supplementary Material online. When performing the test, low-frequency variants (those present in fewer than 5% of the lines for each species) were excluded. Polarized MK tests were performed using *D. yakuba* sequence as an additional outgroup sequence to polarize for lineage-specific substitutions.

## Supplementary Material

Supplementary data are available at *Molecular Biology and Evolution* online. Original data underlying this manuscript can be accessed from the Stowers Original Data Repository at http://www.stowers.org/research/publications/libpb-1330; last accessed October 31, 2018.

## Supplementary Material

Supplementary Data
