# Supplementary material for: Functional Consequences of the Evolution of Matrimony, a Meiosis-Specific Inhibitor of Polo Kinase
Source: Mol Biol Evol. 2018 Oct 23;36(1):69–83. doi: 10.1093/molbev/msy197 (PMC6340472; doi:10.1093/molbev/msy197)
Supplement: Supplementary Data [file msy197_supp.zip › Supplemental_figure_legends_MBE-18-0583.R1.pdf]

SUPPLEMENTAL FIGURE LEGENDS

Figure S1. Multiple sequence alignment of Mtrm homologs. The LEN degron motif region, S/TP region, SAM-proximal region, and SAM domain are highlighted in the schematic above the alignments, and fully conserved residues are highlighted using the default Clustal color scheme. Protein lengths are shown at the end of each alignment. The GUIDANCE2 confidence scores (see Materials and Methods) for each column in the alignment are shown below the MSA. Black columns indicate those above the threshold of 0.93, while white columns are those below the threshold.

Figure S2. Western blot of FLAG-Mtrm protein levels in late-stage oocytes.

Figure S3. Graph of hatch rates, comparing *mtrm* homologs expressed in a wild-type background to their hatch rates in a *polo/*+ heterozygous background.

Figure S4. Schematic of the three chimeric Mtrm proteins: Mtrm$^{Dwil+LEN}$, Mtrm^{GWG}, and Mtrm^{WGW}.
