# Supplementary material for: Functional Consequences of the Evolution of Matrimony, a Meiosis-Specific Inhibitor of Polo Kinase
Source: Mol Biol Evol. 2018 Oct 23;36(1):69–83. doi: 10.1093/molbev/msy197 (PMC6340472; doi:10.1093/molbev/msy197)
Supplement: Supplementary Data [file msy197_supp.zip › Supplemental_figures_MBE-18-0583.R1.pdf]

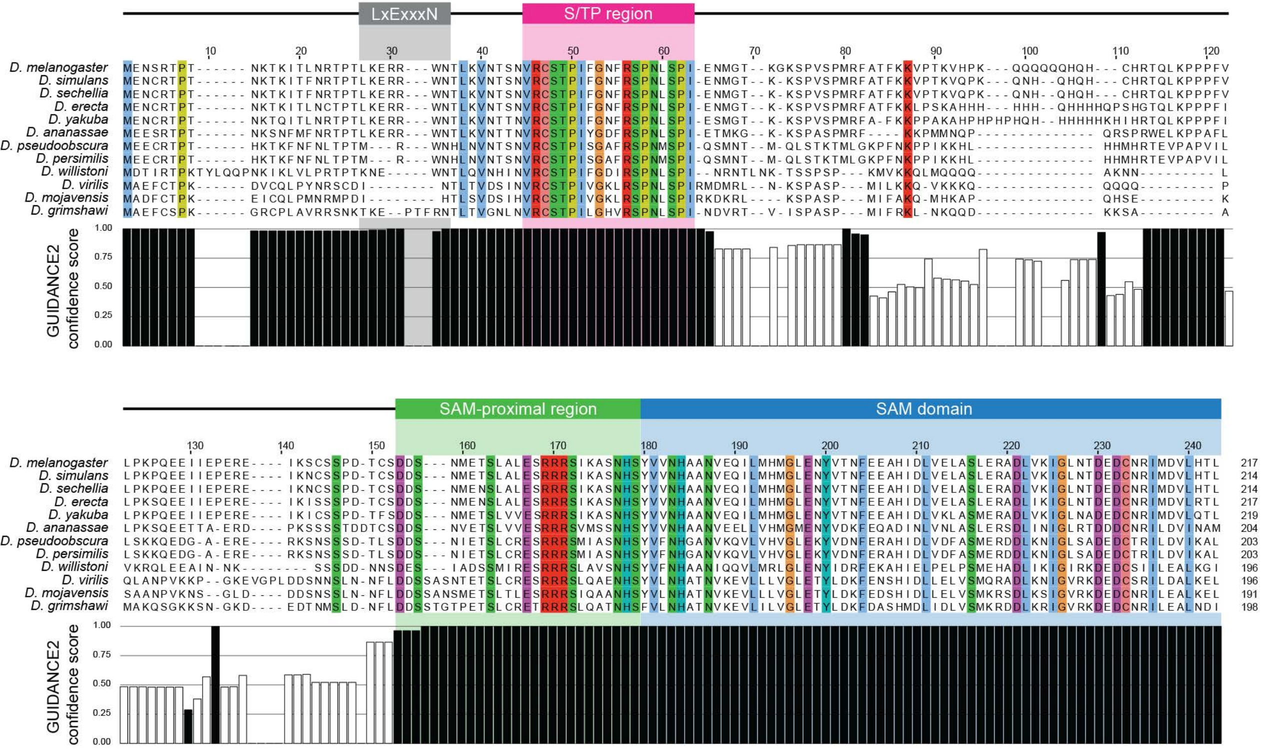
LxExxxN
S/TP region
D. melanogaster
D. simulans
D. sechellia
D. erecta
D. yakuba
D. ananassae
D. pseudoobscura
D. persimilis
D. willistoni
D. virilis
D. mojavensis
D. grimshawi
GUIDANCE2 confidence score
1.00
0.75
0.50
0.25
0.00
SAM-proximal region
SAM domain
217
214
214
217
219
204
203
203
196
196
191
198

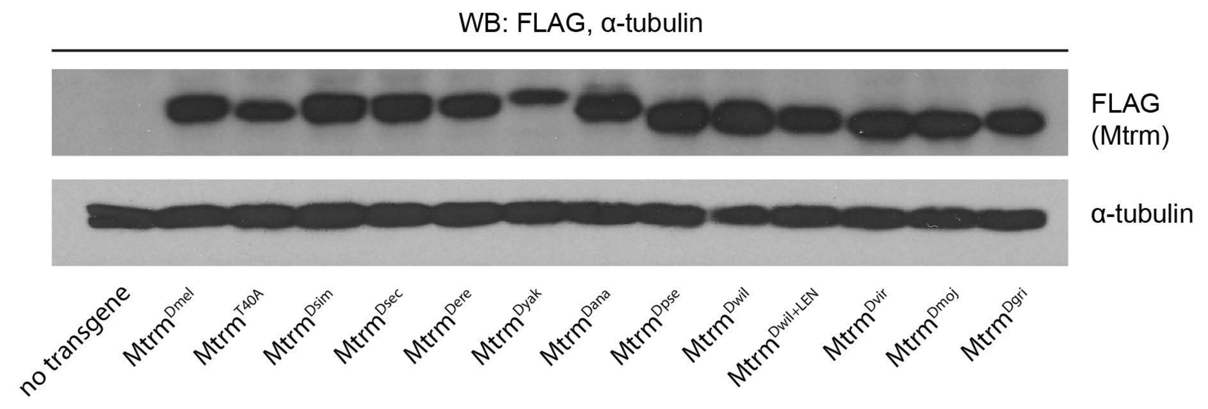
WB: FLAG, α-tubulin
FLAG (Mtrm)
α-tubulin
no transgene
MtrmDmel
MtrmT40A
MtrmDsim
MtrmDsec
MtrmDere
MtrmDyak
MtrmDana
MtrmDpse
MtrmDwil
MtrmDwil+LEN
MtrmDvir
MtrmDmoj
MtrmDgri

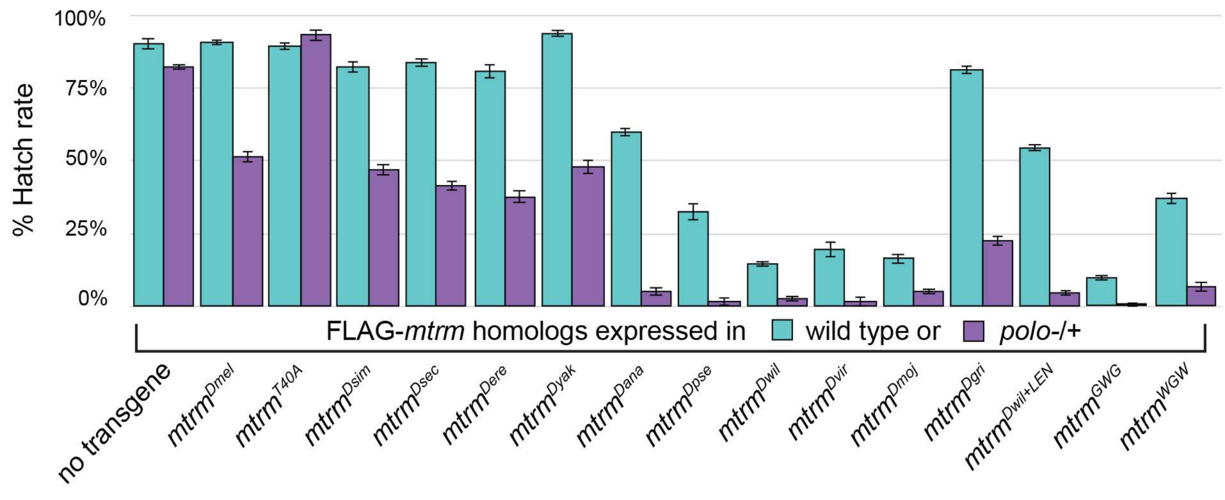

% Hatch rate
100%
75%
50%
25%
0%
FLAG-mtrm homologs expressed in wild type or polo-/+
no transgene
mtrm^Dmel
mtrm^T40A
mtrm^Dsim
mtrm^Dsec
mtrm^Dere
mtrm^Dyak
mtrm^Dana
mtrm^Dpse
mtrm^Dwil
mtrm^Dvir
mtrm^Dmoj
mtrm^Dgri
mtrm^Dwil+LEN
mtrm^GWG
mtrm^WGW

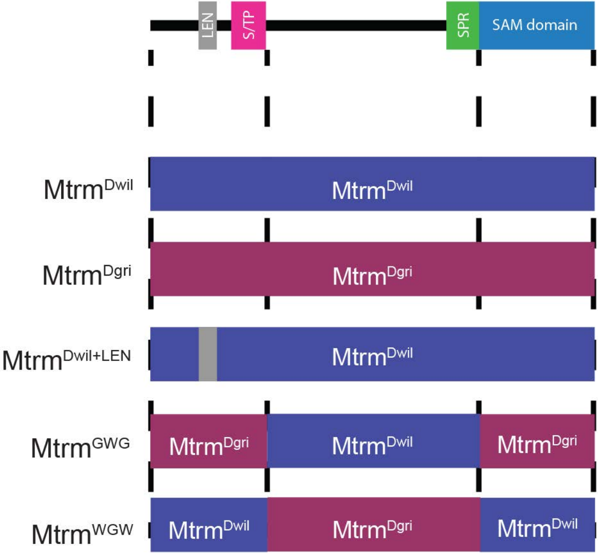

LEN
S/TP
SPR
SAM domain
Mtrm^{Dwil}
Mtrm^{Dwil}
Mtrm^{Dgri}
Mtrm^{Dgri}
Mtrm$^{Dwil+LEN}$
Mtrm^{Dwil}
Mtrm^{GWG}
Mtrm^{Dgri}
Mtrm^{Dwil}
Mtrm^{Dgri}
Mtrm^{WGW}
Mtrm^{Dwil}
Mtrm^{Dgri}
Mtrm^{Dwil}
