# Supplementary material for: Functional Consequences of the Evolution of Matrimony, a Meiosis-Specific Inhibitor of Polo Kinase
Source: Mol Biol Evol. 2018 Oct 23;36(1):69–83. doi: 10.1093/molbev/msy197 (PMC6340472; doi:10.1093/molbev/msy197)
Supplement: Supplementary Data [file msy197_supp.zip › Supplementary_data_MBE-18-0583.R1.pdf]

Supplementary data 1

mtrm* sequences from *Drosophila

>Drosophila_melanogaster_mtrm
ATGGAGAATTCTCGCACGCCCACGAACAAGACCAAAATTACGCTTAATCGCACGCCAACGC
TAAAGGAGCGCAGATGGAACACCCTGAAGGTGAACACCTCCAACGTGCGATGCTCTACTC
CGATCTTTGGCAACTTCCGTTCGCCCAATCTCTCGCCCATCGAGAATATGGGCACGAAGG
GGAAGAGTCCAGTGTCGCCCATGCGGTTCGCTACCTTCAAGAAAGTGCCAACGAAGGTGC
ATCCCAAGCAGCAGCAGCAGCAGCAGCATCAGCACTGCCATCGCACTCAGCTTAAGCCCC
CGCCATTCGTGCTGCCCAAGCCGCAGGAGGAGATCATCGAGCCGGAGCGAGAAATAAAG
AGCTGCAGCAGCCCGGATACCTGTTCGGATGACTCGAATATGGAGACCTCACTGGCCTTG
GAGTCGCGTCGTCGTTCCATCAAAGCATCGAACCACTCGTACGTGGTTAACCATGCCGCC
AATGTGGAACAGATTCTCATGCACATGGGCCTGGAGAACTATGTGACCAATTTCGAAGAGG
CTCACATCGATCTGGTGGAACTGGCATCCTTGGAGCGTGCTGATCTTGTTAAAATCGGCCT
AAATACCGATGAGGATTGCAACCGTATCATGGATGTGCTCCACACTCTT
>Drosophila_simulans_mtrm
ATGGAGAATTGTCGCACGCCCACGAACAAGACCAAAATTACGTTTAATCGCACGCCAACGT
TAAAGGAGCGCAGATGGAACACCCTGAAGGTGAACACCTCCAACGTGCGATGCTCTACTC
CGATCTTCGGCAACTTCCGTTCGCCCAATCTCTCGCCCATCGAGAATATGGGCACGAAGAA
GAGTCCAGTGTCGCCCATGCGGTTCGCCACCTTCAAGAAAGTGCCAACGAAGGTGCAGCC
CAAGCAGAACCATCAGCATCAGCACTGCCATCGCACTCAGCTTAAGCCCCCGCCATTTGT
GCTGCCCAAGCCGCAGGAGGAGATCATCGAGCCGGAGCGAGAAATAAAGAACTGCAGCA
GCCCGGACACCTGTTCGGATGACTCGAATATGGAGACCTCACTGGCCTTGGAGTCGCGTC
GTCGTTCCATCAAAGCATCGAACCACTCGTACGTGGTGAACCATGCCGCCAATGTGGAAC
AGATTCTCATGCACATGGGCCTGGAGAACTATGTGACCAATTTCGAAGAGGCTCACATCGA
TCTGGTGGAACTGGCATCCTTGGAGCGTGCTGATCTTGTCAAAATCGGCCTAAATACCGAT
GAGGATTGCAACCGCATCATGGATGTGCTCCACACTCTT
>Drosophila_sechellia_mtrm
ATGGAGAATTGTCGCACGCCCACGAACAAGACCAAAATTACGTTTAATCGCACGCCAACAT
TAAAGGAGCGCAGATGGAACACCCTGAAGGTGAACACCTCCAACGTGCGATGCTCTACTC
CGATCTTCGGCAACTTCCGTTCGCCCAATCTCTCGCCCATCGAGAATATGGGCACGAAGAA
GAGTCCAGTGTCGCCCATGCGTTTCGCCACCTTCAAGAAAGTGCCAACGAAGGTGCAGCC
CAAGCAGAACCATCAGCATCAGCACTGCCATCGCACTCAGCTTAAGCCCCCGCCATTCGT
GCTGCCCAAGCCGCAGGAGGAGATCATTGAGCCGGAGCGAGAAATAAAGAACTGCAGCA
GCCCGGACACCTGTTCGGATGACTCGAATATGGAGAACTCACTGGCCTTGGAGTCGCGTC
GTCGTTCCATTAAAGCATCGAACCACTCGTACGTGGTCAACCATGCCGCCAATGTGGAACA
GATTCTCATGCACATGGGCCTGGAGAACTATGTGACCAATTTCGAAGAGGCTCACATCGAT
CTGGTGGAACTGGCATCCTTGGAGCGTGCTGATCTTGTCAAAATCGGCCTAAATACCGATG
AGGATTGCAACCGCATCATGGATGTGCTCCACACTCTT
>Drosophila_mauritiana_mtrm
ATGGAGAATTGTCGCACGCCCACGAACAAGACCAAAATTACGTTTAATCGCACGCCAACGT
TAAAGGAGCGCAGATGGAACACCCTGAAGGTGAACACCTCCAACGTGCGATGCTCTACTC
CGATCTTCGGCAACTTCCGTTCGCCCAATCTCTCGCCCATCGAGAATATGGGCACGAAGAA
GAGTCCAGTGTCGCCCATGCGGTTCGCCACCTTCAAGAAAGTGCCAACGAAGGTGCAGCC
CAAGCTGAACCATCAGCATCAGCACTGCCATCGCACTCAGCTTAAGCCCCCGCCATTCGT
GCTGCCCAAGCCGCAGGAGGAGATCATCGAGCCGGAGCGAGAAATAAAGAACTGCAGCA
GCCCGGACACCTGTTCGGATGACTCGAATATGGAGACCTCACTGGCCTTGGAGTCGCGTC
GTCGCTCCATCAAAGCATCGAACCACTCGTACGTGGTGAACCATGCCGCCAATGTGGAAC
AGATTCTCATGCACATGGGCCTGGAGAACTATGTGACCAATTTTGAAGAGGCTCACATCGA

TCTGGTGGAATTGGCATCCTTGGAGCGTGCTGATCTTGTCAAAATCGGCCTAAATACCGAT
GAGGATTGCAACCGCATCATGGATGTGCTCCACACTCTT
>Drosophila_erecta_mtrm
ATGGAGAATTGTCGTACGCCCACGAACAAGACGAAAATTACTTTGAATTGCACGCCAACGT
TAAAGGAGCGCAGATGGAACACCCTGAAGGTGAACACCTCCAACGTGCGATGCTCTACTC
CGATTTTCGGCAACTTCCGTTCGCCCAATCTTTCGCCCATTGAGAATATGGGCACAAAGAA
GAGTCCAGTGTCGCCCATGCGGTTCGCCACCTTCAAGAAACTGCCATCGAAGGCGCATCA
CCATCACCATCATCAGCACCATCACCATCAACCCAGTCATGGCACTCAGCTGAAGCCGCC
GCCTTTTATACTGCCCAAGTCGCAGGAGGAGATCATCGAGCCAGAGCGAGAAATTAAGAT
CTCCAGTAGCCCGGACACCTGTTCGGACGACTCGAATATGGAGAACTCACTGGCCTTGGA
GTCGCGTCGGCGTTCCATCAAGGCATCGAACCACTCGTATGTTGTTAACCATGCCGCCAAT
GTGGAACAGATACTTATGCACATGGGCTTGGAGAACTATGTGACCAACTTCGAAGAGGCTC
ACATCGATCTGGTGGAACTGGCGTCTTTGGAGCGTGCGGATCTTATCAAAATTGGCCTAAA
TACCGATGAGGATTGTAACCGCATCATGGATGTGCTCCGCACCCTT
>Drosophila_orena_mtrm
ATGGAGAATTGTCGTACGCCCACGAACAAGACTAAAATAACTTTGAATTGCACGCCAACGT
TAAAGGAGCGCAGATGGAACACCCTGAAGGTGAACACCTCCAACGTGCGATGCTCTACTC
CGATTTTCGGCAACTTCCGTTCGCCCAATCTTTCGCCCATCGAGAATATGGGTACAAAGAA
GAGTCCAAAGTCGCCCATGCGGTTCGCCACGTTCAAGAAACTGCCAACGAAGGCGCATCA
CCATCCTCAGCATCATCACCATCACCATCACCATCAAGCCAGTCATGGCACTCAGCTGAAG
CCGCCGCCTTTTATACTGCCCAAGCCGCAGGAGGAAATCATCGAGCCGGAGCGAGAAATA
AAGGTCTCCAGTAGCCCGGATATGGAGCCCGGATCCTGTTCGGATGACTCGAATATGGAG
AACTCACTGACCTTGGAGTCGCGTCGGCGTTCCATCAAAGCATCGAACCACTCGTACGTTG
TTAACCATGCCGCCAATGTGGAACAGATACTTATGCGCATGGGCTTGGAGAACTATGTGAC
CAACTTTGAAGAGGCTCACATCGATCTGGTGGAACTGGCTTCCTTGGAGCGTGCGGATCTT
GTAAAAATTGGCCTAAATACCGATGAGGATTGCAACCGCATCATGGATGTGCTCCGCACCC
TT
>Drosophila_yakuba_mtrm
ATGGAGAATTGTCGTACGCCCACGAACAAGACCCAAATTACGTTGAATCGCACGCCAACGT
TAAAGGAGCGCAGATGGAACACCCTCAAAGTGAACACCACCAACGTGCGATGCTCTACGC
CGATTTTCGGAAACTTCCGTTCGCCCAATCTTTCGCCCATCGAGAGCATGGGCACCAAGAA
GAGTCCAGTTTCGCCCATGCGGTTTGCCTTTAAGAAACCGCCAGCGAAGGCGCATCCCCA
TCCACATCCCCATCAGCACCATCACCATCACCATAAGCACATTCATCGCACGCAGCTGAAG
CCGCCGCCATTTATACTGCCCAAGCCGCAGGAGGAGATCATTGAGCCGGAGCGAGAAATA
AAGATCTGCAGCAGCCCGGACACCTTTTCAGATGACTCGAATATGGAGACCTCACTGGTC
GTGGAGTCGCGTCGCCGTTCTATCAAAGCATCGAACCACTCGTACGTTGTGAACCATGCT
GCCAATGTGGAACAGATACTTATGCACATGGGCTTGGAGAACTATGTGACCAACTTCGAAG
AGGCACACATCGATCTGGTGAAACTGGCGTCCATGGAGCGTGCTGATCTGGTCAAAATCG
GTCTAAATGCCGATGAGGATTGCAATCGCATCATGGATGTGCTCCAAACTCTT
>Drosophila_teissieri_mtrm
ATGGAGAATTGTCGTACGCCCACGAACAAGACCAAAATTACCTTGAATTGCACGCCAACGT
TAAAGGAGCGCAGATGGAACACCCTCAAAGTGAACACCACCAACGTGCGTTGCTCTACGC
CGATTTTCGGCAACTTCCGTTCGCCCAATCTGTCGCCCATCGAGAACATGGGCACCAAGAA
GAGTCCAGTGTCGCCCATGCGGTTCGCCTTTAAGAAACCGCCAGCGAAGGCGCATCAACA
TCAGCATCAGCATCAGCACCATCACCATAAGCACAGTCATCGCACTCAGCTGAAGCCGCC
GCCATTTATACTGCCCAAGCCGCAGGAGGAGATCATTGAGCCGGAGCGAGAAATAAAGAT
CTGCAGCAGCCCGGACACCTTTTCAGATGACTCGAATATGGAGACCTCACTGGTCGTGGA
GTCGCGTCGGCGTTCTATCAAAGCATCGAACCACTCGTACGTTGTGAACCATGCCGCCAAT
GTGGAACAGATACTTATGCACATGGGCTTGGAGAACTATGTGTCCAACTTCGAAGAGGCTC
ACATCGATCTGGTGAAACTGGCCTCCATGGAGCGTGCTGATCTTGTCAAAATCGGTCTAAA
TGCCGATGAGGATTGCAACCGCATCATGGATGTGCTCCACACTCTT

```
>Drosophila_santomea_mtrm
ATGGAGAATTGTCGTACGCCCACGAACAAGACCCAAATTACGTTGAATTGCACGCCAACGT
TAAAGGAGCGCAGATGGAACACCCTCAAAGTGAACACCACCAACGTGAGATGCTCTACGC
CGATTTTCGGAAACTTCCGTTCGCCCAATCTGTCACCCATCGAGAGCATGGGCACCAAGAA
GAGTCCAGTGTCGCCCATGCGGTTCGCCTTTAAGAAACCGCCTGCGAAGGCGCATCCCCA
TCCCCATCCCCATCAGCACCATCACCATCACCATAAGCACATTCATCGCACGCAGCTGAAG
CCGCCGCCATTTATACTGCCCAAGCCGCAGGAGGAGATCATTGAGCCGGAGCGAGAAATA
AAGATCTGCAGCAGCCCGGACACCTTTTCAGATGACTCGAATATGGAGACCTCACTGGTC
GTGGAGTCGCGTCGGCGTTCCATCAAAGCATCGAACCACTCGTACGTTGTGAACCATGCC
GCCAATGTGGAACAGATACTTATGCACATGGGCCTGGAGAACTATGTGACCAACTTCGAAG
AGGCTCACATCGATCTGGTGAAACTGGCCTCCATGGAGCGGGCTGATCTTGTCAAAATCG
GTCTAAATGCCGATGAGGATTGCAACCGCATCATGGATGTGCTCCACACTCTT
>Drosophila_eugracilis_mtrm
ATGGAGAATTACCGTACACCCATAAACGAAACGAAAATCGCCTTTAACCGCACTCCAACGT
TAAAGGAGCGTAAGTGGAACACCCTCAAGGTTAACACCTCCAATGTACGATGCTCAACTCC
AATTTTTGGGAATTTTCGTTCACCTAATCTCTCGCCCATCGAGAGCATGTGCATGAAGAAGA
GTCCGGTATCGCCCATGCGGTTTGAAACATTAATGAAGCCGCCGATGAAGGTCCATCATCA
TCGTCATCAACGTCAACATCATCAACATATTCACCGTACACAGCTAAAACCTCCGGTTTTTA
ACTTGCCCAAACCACAGGAGGAGGTTATTGAGCCGGAGCGGGAATTCAAAAGTTGCAGCA
GTCCGGACACCTGTTCGGATGACTCAAATATGGAGACCTCGCTCACATTGGAGACGCGTC
GCCGTTCTATAAAAGCCTCCAACCATTCTTATGTAGTAAACCATGCTGCCAATGTGGAACA
GGTACTTATGCACATGGGCCTGGAGAACTATGTGACCAATTTTGAAGAGGCCCATATCGAT
TTGGTGGAACTGGCTTCCATGGAGCGTGCGGATCTCATGAAAATCGGCCTTAAAGCTGAA
GAAGACTGTAACCGAATAATGGATGCCCTTCAAACTCTA
>Drosophila_biarmipes_mtrm
ATGGAGAATTGCCGTACACCCACAAATAAGGTCAAAATTACCTTTCCCAGGACTCCAACGC
TAAAGGAACGCAGATGGAACACGCTGAAGGTTAACACCTCCAACGTGCGCTGCTCCACAC
CGATTTTCGGCAACTTCCGTTCGCCCAATCTCTCGCCCATCGAGAACATGGGCTCGAAGAA
GAGCCCTGCATCGCCCATGCGGTTCGACGCCTTCAAGAAGCCACCGATGAAGACTGTTCA
TCATCAACATAGCCATCAGCTTAAACCACCGGTCTTCAATTTGCCGAAAGCGCATGAGGAG
ATTACCGAGCCGGAGCGGGAGATCAGGAGCTGCAGCAGCCCGGACACCTGTTCGGATGA
CTCGAGTAGGGATACCTCGTTGACCTTGGAGTCGCGTCGCCGTTCCATAAAGGCCTCCAA
CCACTCGTACGTAGTGAATCATGCTGCCAATGTGGAGCAGATCCTCATGCACATGGGCCT
GGAGAACTATGTGACCAACTTCGAGGAGGCTCATATCGATCTGGTGGAACTGGCCTCCAT
GGAACGGGCTGATCTCATCAAAATTGGCCTAAACACGGATGAGGATTGCAACCGCATCAT
GGATGTCCTCCACACTCTC
>Drosophila_suzukii_mtrm
ATGGAGAATTGTCGTACACCCACAAACAAGGTCAAAATTACCTTTCCCCGGACTCCAACGC
TAAAGGAGCGCAGATGGAATACCCTGAAGGTTAACACCTCCAACGTGCGATGCTCCACAC
CGATTTTCGGCAACTTCCGTTCGCCCAATCTATCGCCCATCGAAAACATGGGCACGAAGAA
GAGTCCGGCATCGCCCATGCGGTTCGAAGCATTCAAGAAGCCGCCAATGAAAATTGCTCA
TAATCAACATAGTCATCAGCATAGTCACCGCACTCAGCTCAAACCACCGGTCTTCAATTTGC
CCAAGCCGCAGGAAGAGATTACCGAGACGGAGAGTGAGATTAAGAGCTGCAGCAGCCCG
GACACCTGTTCGGATGACTCCAGCAGGGATACCTCGTTGACTTTGGAGTCGCGTCGTCGT
TCCATAAAGGCGTCAAACCATTCGTACGTAGTTAACCATGCTGCCAATGTGGAGCAGATTC
TCATGCACATGGGCCTGGAGAATTATGTGACCAACTTCGAGGAGGCTCACATCGATCTAGT
GGAACTGGCCTCCATGGAACGGGCTGATCTCATTAAAATTGGCCTAAACACCGATGAGGAT
TGCAACCGTATCATGGATGTTCTCCACACTCTC
>Drosophila_takahashii_mtrm
ATGGAGAATTGTCGTACACCCACAAACAAGACCAAAATTACCTTTAACCGGACTCCAACGT
TAAAGGAGCGCAGATGGAACACTCTGAAGGTGAACACCACCAATGTGCGCTGCTCCACTC
```

CGATTTTCGGCAACTTCCGTTCGCCGAATCTCTCGCCCATCGAGAATATGGGCACGAAGAA
GAGTCCTGCTTCTCCCATGCTCTTCAAGAAGCCGCCGCTGAAGGTACACCATCAACAGCAA
CACCACCAGCATATTCATCGCAATCACCTCAAACCGCCGGTCTTCAATTTGCCCAAAGCGC
AGGAGGAGATCATCGAGCCGGAGCGGGAATCCAAGAGCTGCAGCAGCCCGGACACCTGT
TCGGATGACTCCAATATGGAGACCTCGATGGCCCGGGAGTCGCGTCGCCGTTCCATAAAG
GCATCCAACCATTCGTATGTAGTTAACCATGCCGCCAATGTGGAACAGATTCTCATGCACA
TGGGCCTGGAGAACTATGTGACCAATTTCGAGGAGGCTCACATCGACCTGGTGGAACTGG
CCTCCATGGAGCGGGCTGATCTCATCAAAATTGGCCTAAACACCGATGAGGATTGCAACC
GCATTATGGATGTCCTCCACACTCTT
>Drosophila_ficusphila_mtrm
ATGGAAAATTCTCGCACACCCGCAAAAAAGACCACAATAACGCTTAACCGCACTCCAACGT
TGAAGGAGCGCAGGTGGAACACACTGAAGGTGAACACCACCAATGTGCGCTGCTCCACAC
CGATTTTCGGCAACTTCCGTTCGCCTAACCTATCCCCCATCGAGAACATGGGCACTAAGAA
GAGTCCGGTATCACCCATGCGGTTTGCAAACATCAAAAAGCCGCCGATGAAGATGCATTCT
CAGCATTATCAAAAAAAACACCAGCACGAGCAAAGCTATCGCACTCAGCTTAAGCCACCGG
TCTTTCGTTTACCCAAACCGCAGGAGGAGATTGACGACCCAGTGTCGGAAATTAAGATCAA
TAGCAGTCCGGACACCTGTTCCGATGATTCGAATGTGGACACTTCGTTGACCCTGGAGTC
GCGTCGCCGTTCCATAACGGCTTCTAACCATTCATACGTAGTTAACCATGCCGCTAATGTG
GAACAGATTCTCGTGCACATGGGCCTGGAGAAATACGTGACCAACTTCGAGGAGGCCCAC
ATCGATCTCGTGGAACTGGCTTCAATGAAGCGGGATGATCTCATTAAAATTGGCTTGAACA
CCGATGAGGACTGTTACCGCATTTTGGATGCCCTT
>Drosophila_rhopaloa_mtrm
ATGGAGAATTGCCTTACACCCACAAACAAGACAAAAATTACTTTTAACCGCACTCCAACTTT
AAAAGAGCGCAGGTGGAACACCCTAAGGGTGAACACCTCCAATGTGCGGTGCTCTACGCC
TATATACGGGAACTTCCGTTCGCCCAATCTCTCGCCCATCGAGAATATGGGCACGAAAAAG
AGTCCGGCATCGCCCATGCTGTTTGGAGCCTTCAAGAAGCAGCCGATGAAGATGCATCAT
CAGCAGCACCACCAGTATAATCCTCGCAATCAGTTGAAACCCCCGATTTTCAATCTGCCCA
AACCGCAGGAGGAGATTAAGGAGCCGGAGCGAGAAATAAAGAGCAGCAGCAGCCCGGAA
ACCTGTTCGGATGACTCAAATATGGAGACCTCGTTGACCTTGGAGTCGCGTCGTCGTTCCA
TTATAGCTTCAAACCACTCGTACGCTGTAAACCATGCCGCCAATGTGGAGCAAATTCTCAT
GCATATGGGTCTGGAGAACTATGTGACCAACTTCGAGGAAGCCCACATTGATCTGGTAGAA
CTGGCCTCCATGAAGCGGGCTGATCTTATCAAAATTGGCCTAAACACTGATGAGGATTGCA
ACCGCATCATGGAAGTTCTTGATACTCTC
>Drosophila_elegans_mtrm
ATGGAGACCTGTTGCACACCGAAAAACAAAACGAAAATTATCCTTAATCGCACGCCAATTTT
AAAAGAGCGCAGGTGGAACACCTTGAATGTGAACACCTCCAATGTGCGATGCTCGACGCC
AATATTTGGCAACTTTCGATCGCCCAATCTCTCGCCCATCGAGGATATGGGAGTGAAAAAA
AGTCCGGTGTCGCCCATGCAGTTCGGAAACTTTAAGAAGCCGCCGATGCAAATGCATCATT
ATCATCATCATCATCATCAGCAGCACAACCAGCATAGTGGACGCACTCATCTAAAACCCCC
GGTTTTCAACTTGCCGAAGGATGCGAACGAGCCAGACCGCGAATTTAAGAGCAGCAGCAG
CCCGGACACATTTTCGGATGACTCCAATATGGAGACCTCGTTGACCTTGGAGTCGCGTCG
CCGTTCGATAACAGCCTCCAACCACTCGTACGCCGTAAACCATGCCGCCAATGTGGAGCA
GATTCTCATGCACGTGGGCCTGGAGAACTATGTGACCCACTTTGAGGAGGCCCACATCGA
TCTGGTGAAACTGGCCTCCATGGAGCGGGCTGATCTAATCAAAATTGGATTAAAAACCGAT
GAGGATTGCAACCGCATCATGGATGTTCTGCATAACCTT
>Drosophila_kikkawai_mtrm
ATGGAGCACTGCCGTACTCCAACGAACATGAAAAACTACACCTTCAACCGCACTCCAACGA
TGAAGGAGCGGCGATGGAACACCCTGAATGTGAGCAACACCAGCGTGCGCTGCTCCACG
CCCATATTCGGGGACTTTCGATCGCCCAATCTCTCGCCGATCGTGGGCATGGGTTCGAAG
AAAAGCCCGGTGTCGCGTTTCGAAGAATTGAAGAAGCCGCCGATTAAGCATCTACATCACC
ATCAGAAGCGCAACCCCAAGATGATCAAGCGCAGCGAACTGAAGCCTCCGATTTTCGTCC

TGCCCCAGCCGCAGGAAGAGGTTATGGAGCAGAAGCAACGGGAACTGGAACGGGAGAGG
AAGAGCAACAGCAACAGCAGCCTGGATATCGGTTCGGATGACTCTAATTTGGAGACCTCC
CTCACTGTGGAGTCACGTCGCCGCTCCGTCACAGCCTCCAATCACTCGTATGTGGTGAAC
CATGCCCGCAACGTGGAGCAGGTCCTCGTGCACATCGGCCTGGAGAGCTATGTCCGTCAC
TTTGAGAAGGCCCACATCGATATGGTGACCCTGGCCTCCATGGGACGTGATGATCTGGCC
AAAATTGGCCTCAAGGCCGATGAGGATTGTAATAAGATTATGGATGCCATCAATGCTCTA
>Drosophila_serrata_mtrm
ATGGAGCACTGCCGTACTCCGACCAACAAGAAAAACTACACCTTCAATCGCACTCCAACGA
TGAAGGAGCGGCGGTGGAACACCCTGAACGTGAGCAACACCAGCGTTCGCTGCTCCACG
CCCATCTTTGGGGACTTCCGATCGCCCAATCTCTCGCCGATCGAGGGCATGGGTCCGAAG
AAAAGTCCCGTGTCACCCATGCGCTTTGAAGCATTCAAGAAGCCGCCAATGAAGTTGGATA
TACATCAGCCGCAAAAGCACCACAATAAGATCATAAATCGCACGGAGCTAAAGCCTCCGAT
TTTCGTACTGCCCCAGCCGCAGGAAGAGATTATGGAGCATAAACAATGGGAATCGGAACG
TGAAAAGGAGATGAAGAGCAATAGCAGCAGCAGCCCGGATACCTGTTCAGATGACTCCAA
TTTGGAGACCTCCCTAACCGTGGAGTCACGCCGCCGCTCCATTACAGCCTCCAATCACTC
GTATGTGGTGAATCATGCCCGAAATGTGGAGCAGGTACTTGTGCACATTGGTCTGGAGAA
CTATGTCCGTCACTTTGAGAAGGCTCACATTGATTTGGTGACCCTGGCCTCCATGGACGC
GAGGATCTGGCCAAAATTGGCCTCAACGCCGATGAGGATTGCAATCGCATTATGGATGCC
ATCAATGCTCTT
>Drosophila_bipectinata_mtrm
ATGGAGGAGTACCGTACACCGACCAATAAATCCAATTTCACCTTCAATCGAACACCAACTC
TGAAGGAACGGCGCTGGAATACCCTGAAAGTGAACACCACCAATGTGCGCTGTTCAACGC
CCATTTACGGCGATTTTCGTTCGCCAAATCTGTCGCCGATTGAAAGTATGAAGGCCAAGAA
GAGTCCGGCTTCCCCCATGTACTTCAAGAAGCCAACAACAAACCAACCACAAAAAAGTCCA
CGCTGGGAGCTCAAGCCGCCGGTATTTCTTTTGCCAAAATCGCACGAGGAGATCACCGCC
GAAAAGGAGCCGAAGAGCAGCAGCAGTACGGATGATACCTGCTCGGATGACTCGAATGTG
GAGACTTCCCTGGTCGTGGAGTCCCGTCGTCGGTCAGTTATGTCATCCAACCACTCATACG
TGGTGAATCATGCCGCCAATGTGGAGGAACTGCTCGTCCACATGGGCATGGAGAACTACG
TGGATAAATTTGAACAGGCCGACATCAATCTGGTCAACCTGGCCTCCTTGGAACGTTCGGA
TCTGATCAACATAGGAATGCGCACCGATGATGATTGTAATCGCATTTTAGATGTCATCAACG
CTATG
>Drosophila_ananassae_mtrm
ATGGAGGAGTCCCGTACACCAACCAATAAATCCAACTTTATGTTCAACCGAACGCCCACTT
TGAAGGAACGGCGCTGGAATACTCTGAAAGTGAACACCACCAATGTGCGCTGTTCCACAC
CTATTTACGGCGACTTTCGTTCGCCAAATCTGTCACCCATTGAGACCATGAAGGGCAAAAA
GAGTCCGGCTTCACCCATGCGTTTCAAAAAGCCAATGATGAACCAACCGCAAAGAAGTCCA
CGCTGGGAGCTTAAGCCGCCCGCATTTCTTTTGCCAAAATCGCAGGAGGAGACTACCGCC
GAAAGGGATCCCAAGAGCAGCAGCAGTACGGATGATACCTGCTCGGATGACTCGAATGTG
GAGACTTCTCTGGTCGTGGAGTCACGTCGTCGCTCCGTCATGTCGTCCAACCACTCGTAC
GTGGTGAATCATGCCGCCAATGTGGAGGAACTGCTCGTCCACATGGGCATGGAGAACTAC
GTGGATAAGTTTGAGCAAGCCGACATCAATCTGGTTAACCTGGCCTCCTTGGAACGTTCTG
ATCTGATCAACATAGGACTGCGCACCGATGATGACTGCAATCGCATTTTAGATGTCATCAA
CGCCATG
>Drosophila_pseudoobscura_mtrm
ATGGAGGAATGCCGCACACCGACGCACAAAACCAAATTCAACTTTAATCTCACTCCCACGA
TGAGGTGGAACCACCTGAATGTGAACACATCGAATGTGCGTTGCTCCACTCCGATCTCTGG
CGCTTTCCGATCGCCCAACATGTCGCCCATCCAGAGTATGAATACCATGCAGCTTTCCACG
AAGACCATGCTGGGGAAGCCCTTCAACAAGCCTCCGATCAAGAAACATTTGCATCATATGC
ACCGAACGGAGGTCCCAGCTCCAGTCATCCTTCTATCCAAGAAGCAGGAAGATGGCGCCG
AGCGGGAGCGTAAGAGCAACAGCAGTTCGGATACCCTCTCCGACGACTCGAATATTGAGA
CCTCATTGTGCCGCGAATCGCGTCGTCGCTCCATGATAGCCTCCAATCACTCGTACGTGTT

CAATCATGGGGCCAATGTGAAACAAGTCCTCGTGCATGTTGGTCTGGAGAAATATGTGGAT
AACTTCGAGAGGGCTCACATTGATCTGGTCGATTTTGCCTCCATGGAGAGGGACGATTTAA
AAAACATTGGCCTGTCCGCCGATGAGGATTGCACTCGCATTCTAGACGTTATAAAAGCCCT
C
>Drosophila_persimilis_mtrm
ATGGAGGAATGCCGCACACCGACGCACAAAACCAAATTCAACTTTAATCTCACTCCCACGA
TGAGGTGGAACCACCTGAATGTGAACACATCGAATGTGCGTTGCTCCACTCCGATCTCTGG
CGCTTTCCGATCGCCCAACATGTCGCCCATCCAGAGTATGAATACCATGCAGCTTTCCACG
AAGACCATGCTGGGGAAGCCCTTCAACAAGCCTCCGATCAAGAAACATTTGCATCATATGC
ACCGAACGGAGGTCCCAGCTCCAGTCATCCTTCTATCCAAGAAGCAGGAAGATGGCGCCG
AGCGGGAGCGTAAGAGCAACAGCAGTTCGGATACCCTCTCCGACGACTCGAATATTGAGA
CCTCATTGTGCCGCGAATCGCGTCGTCGCTCCATGATAGCCTCCAATCACTCGTACGTGTT
CAATCATGGGGCCAATGTGAAACAAGTCCTCGTGCATGTTGGTCTGGAGAAATATGTGGAT
AACTTCGAGAGGGCTCACATTGATCTGGTCGATTTTGCCTCCATGGAGAGGGACGATTTAA
AAAACATTGGCCTGTCCGCCGATGAGGATTGCACTCGCATTCTAGACGTTATAAAAGCCCT
C
>Drosophila_willistoni_mtrm
ATGGATACCATACGCACACCGAAAACTTACTTGCAACAGCCAAATAAGATTAAACTTGTCCT
GCCACGCACACCAACCAAAAATGAATGGAATACCCTGCAAGTGAACCACATCAATGTGCGT
TGCTCTACGCCCATTTTTGGTGATATTCGTTCACCCAACTTATCGCCCATTAACCGTAATAC
ACTTAACAAAACATCATCCCCATCCCCCAAGATGGTCAAGAAGCAATTAATGCAGCAGCAG
CAGGCCAAGAACAACTTGGTCAAACGCCAGCTAGAAGAGGCCATCAACAACAAAAGCAGT
TCCGATGACAATAATTCGGATGAATCAATTGCGGACTCTTCAATGATCAGAGAATCCCGCC
GCCGTTCCCTGGCTGTTTCCAATCATTCATATGTATTCAATCACGCGGCCAATATACAGCAA
GTCCTTATGCGTTTGGGTCTCGAAAACTATATCGACAGATTCGAGAAGGCTCACATTGAATT
GCCCGAATTGCCCAGCATGGAGCATGCTGATCTTATAAAGATTGGCATACGCAAAGATGAG
GATTGTAGTATCATACTGGAGGCACTTAAGGGCATC
>Drosophila_virilis_mtrm
ATGGCAGAATTTTGCACTCCAAAAGACGTGTGCCAGTTACCGTATAATCGCAGCTGCGATA
TCAACACGTTGACCGTGGACAGCATCAATGTGCGCTGCTCCACGCCCATTGTGGGCAAAT
TGCGTTCACCGAATTTGTCGCCAATTCGCATGGATATGCGATTGAACAAAAGTCCGGCATC
GCCGATGATACTCAAGAAGCAAGTGAAGAAAAAACAACAGCAACAACAACCACAACTAGCA
AATCCTGTCAAGAAACCCGGCAAGGAGGTGGGCCCGCTCGATGACAGCAACAATAGCTTG
AACAACTTTCTGGATGATTCGAGCGCCAGCAACACGGAAACTTCGCTGTGCCGGGAATCG
CGTCGTCGTTCGCTCCAGGCTGAGAATCATTCGTATGTCCTGAATCATGCGACCAATGTGA
AGGAAGTGCTGCTCCTGGTCGGCCTGGAGACGTATCTGGACAAATTTGAGAACTCGCACA
TTGATCTACTCGAATTGGTGTCCATGCAGCGTGCCGATCTCAAAAATATTGGCGTACGCAA
GGATGAGGACTGCAGCCGCATACTGGATGCGCTCAAGGAGCTC
>Drosophila_mojavensis_mtrm
ATGGCAGACTTTTGCACACCGAAAGAAATTTGTCAATTGCCGATGAATCGTATGCCCGATA
TTCACACTCTTTCCGTGGACAGCATCCATGTGCGCTGCTCCACGCCGATTGTGGGCAAGTT
GCGTTCGCCAAACTTGTCGCCCATCCGAAAGGATAAGCGATTGAAAAGTCCTGCCTCGCC
GATGATCTTTGCGAAGCAAATGCACAAGGCCCCACAGCATTCGGAAAAATCGGCAGCAAAT
CCTGTCAAGAACTCTGGCCTGGACGATGACAGCAACAGCAGCTTGAATAACTTTTTGGATG
ATTCGAGTGCCAACAGTATGGAGACCTCCCTGACCCTGGAGTCGCGTCGTCGTTCCATTC
AAGCGGCCAATCATTCCTATGTGCTGAATCATGCCACGAATGTGAAGGAAGTGCTGCTCCT
TGTCGGCTTGGAAACGTATCTGGATAAGTTCGAGGACTCCCACATAGATTTACTCGAACTG
GTGTCCATGAAGCGATCAGATCTGAAGAGCATCGGCGTACGCAAAGATGAGGACTGCAAT
CGCATACTGGAAGCCCTCAAGGAGCTG
>Drosophila_grimshawi_mtrm

ATGGCGGAATTTTGCTCACCGAAAGGCAGATGCCCATTGGCAGTCAGACGCTCGAACAAG
ACCAAAGAGCCGACCTTTCGGAACACGCTGACCGTGGGAAACCTCAATGTGCGCTGCTCC
ACGCCCATCTTGGGCCACGTGCGATCGCCCAATTTGTCACCCATTAATGATGTGCGCACG
GTGATAAGTCCAGCATCTCCAATGATTTTTAGAAAATTGAATAAACAACAAGATAAAAAGTC
GGCAGCTATGGCAAAGCAATCAGGAAAGAAGAGCAATGGGAAGGATGAAGATACCAACAT
GAGTTTGGACAACTTCTTGGATGACTCAAGTACCGGCACCCCGGAAACATCATTGTGCCG
GGAGACGCGTCGTCGTTCGTTGCAAGCTACGAATCACTCGTTTGTCCTCAATCATGCCACC
AATGTGAAGGAAGTGCTGATCCTTGTCGGCCTGGAGACCTATCTGGATAAATTCGATGCAT
CGCATATGGATCTGATCGATTTGGTGTCCATGAAACGTGATGATCTCAAAAGAATAGGCGT
GCGCAAGGATGAAGACTGCAATCGCATACTAGAAGCCCTCAATGATATC

Supplementary data 2

Codon-optimized and synthetic/chimeric *mtrm* sequences, represented in Figure S4

>mtrm_Dwil
ATGGACACCATCCGCACGCCCAAGACCTACCTGCAGCAGCCCAACAAGATCAAACTGGTG
CTTCCCCGCACGCCAACGAAGAACGAGTGGAACACCCTGCAGGTGAACCACATCAACGTG
CGATGCTCTACTCCGATCTTTGGCGACATCCGTTCGCCCAATCTCTCGCCCATCAACCGCA
ACACCCTGAACAAGACCAGTTCCCCCTCCCCAAAGATGGTGAAGAAGCAGCTGATGCAGC
AGCAGCAGGCCAAGAACAACCTGGTGAAGCGCCAGCTGGAGGAGGCCATCAACAACAAG
TCCAGCAGCGACGATAACAACTCGGATGAGTCGATCGCCGACTCCTCAATGATCCGCGAG
TCGCGTCGTCGTTCCCTGGCCGTGTCGAACCACTCGTACGTGTTCAACCATGCCGCCAAT
ATCCAGCAGGTGCTCATGCGCCTGGGCCTGGAGAACTATATCGACCGCTTCGAAAAGGCT
CACATCGAGCTGCCCGAACTGCCCTCCATGGAGCACGCTGATCTTATCAAAATCGGCATC
CGCAAGGATGAGGATTGCTCCATCATCCTGGAGGCCCTCAAGGGCATCTAA
>mtrm_Dgri
ATGGCCGAGTTCTGCTCCCCCAAGGGCCGCTGCCCCCTGGCCGTGCGCCGCTCCAACAA
GACCAAGGAGCCCACCTTCCGCAACACCCTGACCGTTGGTAACCTCAACGTGCGTTGCTC
CACCCCCATCCTGGGTCACGTGCGCTCCCCCAATCTGTCGCCCATCAACGATGTCCGCAC
CGTGATCTCCCCCGCTAGCCCCATGATTTTCCGTAAGCTGAACAAGCAGCAGGATAAGAA
GTCCGCCGCCATGGCCAAGCAGTCGGGCAAGAAGAGCAACGGTAAGGACGAGGACACCA
ACATGTCCCTCGATAACTTCCTGGACGACTCCTCCACCGGCACCCCCGAGACCAGCCTCT
GCCGCGAGACTCGCCGTCGCTCCCTCCAGGCTACTAACCACTCCTTCGTGTTGAACCATG
CCACTAACGTCAAGGAGGTGTTGATCTTGGTGGGTCTGGAAACCTACCTGGACAAGTTCG
ACGCCTCCCACATGGATCTGATCGATCTGGTGTCCATGAAGCGTGATGACCTCAAGCGCA
TCGGTGTGCGCAAGGACGAGGACTGCAACCGTATTCTGGAAGCCCTGAACGACATCTAG
>mtrm_Dwil+LEN
ATGGACACCATCCGCACGCCCAAGACCTACCTGCAGCAGCCCAACAAGATCAAACTGGTG
CTTCCCCGCACGCCAACGCTAAAGGAGCGCAGATGGAACACCCTGAAGGTGAACCACATC
AACGTGCGATGCTCTACTCCGATCTTTGGCGACATCCGTTCGCCCAATCTCTCGCCCATCA
ACCGCAACACCCTGAACAAGACCAGTTCCCCCTCCCCAAAGATGGTGAAGAAGCAGCTGA
TGCAGCAGCAGCAGGCCAAGAACAACCTGGTGAAGCGCCAGCTGGAGGAGGCCATCAAC
AACAAGTCCAGCAGCGACGATAACAACTCGGATGAGTCGATCGCCGACTCCTCAATGATC
CGCGAGTCGCGTCGTCGTTCCCTGGCCGTGTCGAACCACTCGTACGTGTTCAACCATGCC
GCCAATATCCAGCAGGTGCTCATGCGCCTGGGCCTGGAGAACTATATCGACCGCTTCGAA
AAGGCTCACATCGAGCTGCCCGAACTGCCCTCCATGGAGCACGCTGATCTTATCAAAATC
GGCATCCGCAAGGATGAGGATTGCTCCATCATCCTGGAGGCCCTCAAGGGCATCTAA
>mtrm_GWG
ATGGCCGAGTTCTGCTCCCCCAAGGGCCGCTGCCCCCTGGCCGTGCGCCGCTCCAACAA
GACCAAGGAGCCCACCTTCCGCAACACCCTGACCGTTGGTAACCTCAACGTGCGTTGCTC

CACCCCCATCCTGGGTCACGTGCGCTCCCCCAATCTGTCGCCCATCAACCGCAACACCCT
GAACAAGACCAGTTCCCCCTCCCCAAAGATGGTGAAGAAGCAGCTGATGCAGCAGCAGCA
GGCCAAGAACAACCTGGTGAAGCGCCAGCTGGAGGAGGCCATCAACAACAAGTCCAGCA
GCGACGATAACAACTCGGATGAGTCGATCGCCGACTCCTCAATGATCCGCGAGTCGCGTC
GTCGTTCCCTGGCCGTGTCGAACCACTCGTTCGTGTTGAACCATGCCACTAACGTCAAGG
AGGTGTTGATCTTGGTGGGTCTGGAAACCTACCTGGACAAGTTCGACGCCTCCCACATGG
ATCTGATCGATCTGGTGTCCATGAAGCGTGATGACCTCAAGCGCATCGGTGTGCGCAAGG
ACGAGGACTGCAACCGTATTCTGGAAGCCCTGAACGACATCTAG
>mtrm_WGW
ATGGACACCATCCGCACGCCCAAGACCTACCTGCAGCAGCCCAACAAGATCAAACTGGTG
CTTCCCCGCACGCCAACGAAGAACGAGTGGAACACCCTGCAGGTGAACCACATCAACGTG
CGATGCTCTACTCCGATCTTTGGCGACATCCGTTCGCCCAATCTCTCGCCCATCAACGATG
TCCGCACCGTGATCTCCCCCGCTAGCCCCATGATTTTCCGTAAGCTGAACAAGCAGCAGG
ATAAGAAGTCCGCCGCCATGGCCAAGCAGTCGGGCAAGAAGAGCAACGGTAAGGACGAG
GACACCAACATGTCCCTCGATAACTTCCTGGACGACTCCTCCACCGGCACCCCCGAGACC
AGCCTCTGCCGCGAGACTCGCCGTCGCTCCCTCCAGGCTACTAACCACTCCTACGTGTTC
AACCATGCCGCCAATATCCAGCAGGTGCTCATGCGCCTGGGCCTGGAGAACTATATCGAC
CGCTTCGAAAAGGCTCACATCGAGCTGCCCGAACTGCCCTCCATGGAGCACGCTGATCTT
ATCAAAATCGGCATCCGCAAGGATGAGGATTGCTCCATCATCCTGGAGGCCCTCAAGGGC
ATCTAA
